# Formulation, physicochemical characterization, and antimicrobial evaluation of drug-loaded essential oil microemulsions against Methicillin-resistant *Staphylococcus aureus* -associated diabetic foot ulcer infections

**DOI:** 10.3389/fmicb.2026.1906281

**Published:** 2026-10-02

**Authors:** Disha Pramanick, Natarajan Chandrasekaran

**Affiliations:** Center for Nanobiotechnology, Vellore Institute of Technology, Vellore, India

**Keywords:** antibacterial efficacy, antibiotics, cinnamon, clove, controlled release, eugenol, microemulsion, MRSA

## Abstract

Diabetes Mellitus, a leading endocrine disorder characterized by persistent hyperglycemia, ischemia, and neuropathy, causes ulcerative wounds that are often colonized by multidrug-resistant pathogens, such as Methicillin-resistant *Staphylococcus aureus* (MRSA), which exacerbate wound infections and can lead to complications such as sepsis and necrosis in Diabetic Foot Ulcers (DFU). This study aims to formulate phytochemical-based nanovesicle carriers for potential wound-healing applications to retain the therapeutic effects of antimicrobial compounds with their localized delivery. This investigation develops microemulsions containing Eugenol, Clove, and Cinnamon oils to encapsulate Vancomycin and Methicillin, preserving their antibacterial activity while providing sustained delivery to the site of action. Microemulsions tested for thermodynamic stability, DLS, zeta potential, pH, FTIR, and TEM, showed optimal hydrodynamic size and PDI (within 30 nm and 0.5), a more negative zeta potential of around −19 mV, and an acidic pH. FTIR of blank emulsions against drug modifications confirmed successful drug incorporation, and TEM showed spherical-oval morphology. Antimicrobial testing using AST, MIC, Antibiofilm, Checkerboard, and Time-kill assays showed that drug-loaded emulsions provided measurable *in vitro* antibacterial activity against the tested clinical MRSA isolates, with activity varying by formulation and isolate (Eug-Van in MRSA-CI 1 and Clo-Van in MRSA-CI 2). Meanwhile, for Methicillin formulations, Clo-Met showed measurable inhibition against both tested isolates. Release kinetics revealed that drug-loaded emulsions showed sustained, diffusion-controlled release of the active therapeutics – Vancomycin and Methicillin over 24 h, with slowly increasing concentrations initially, followed by a plateau with maximum release in the case of Eug-Van microemulsion. These results demonstrate the considerable potential of these drug-loaded microemulsions as advanced nanocarrier systems for localized antimicrobial delivery in MRSA-colonized DFU infections.

## Graphical Abstract

**Figure:**
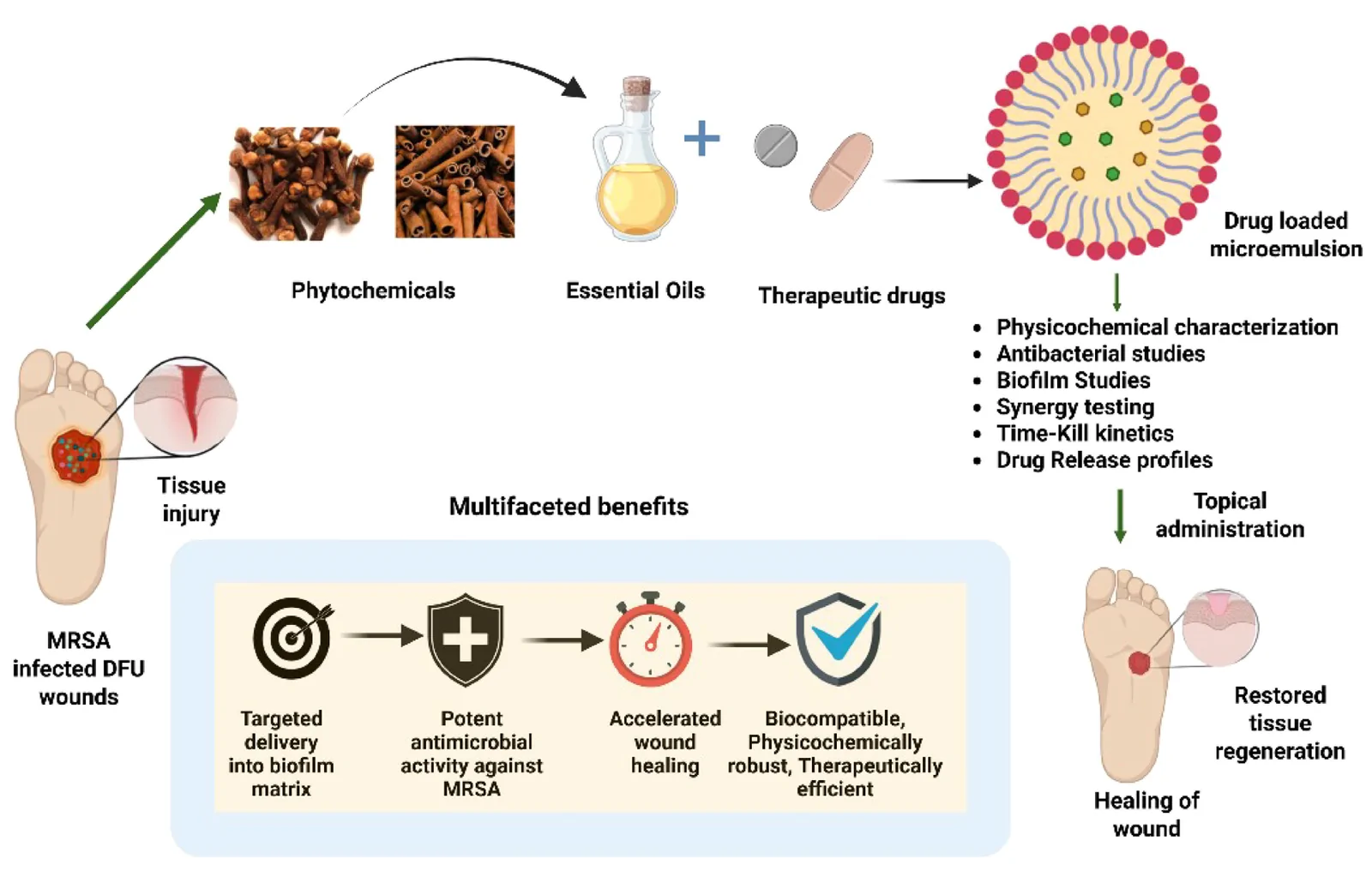
Represents the design of microemulsions and their multifaceted benefits against potential wound infections.

## 1 Introduction

Diabetes Mellitus is recognized as one of the most prevalent and globally consequential metabolic disorders in recent times and is characterized by persistent hyperglycemia along with complications like peripheral neuropathy and ischemia, which affect the quality of life in such patients (Salama et al., 2026). According to data from the International Diabetes Federation (IDF) 2025, the proportion of individuals with diabetes worldwide is predicted to reach 853 million by 2050, making it the largest epidemic of the 21st century (Deng et al., 2023). Diabetes-associated wounds are among the most complicated conditions to treat (Nagaiah et al., 2025) because of features such as diminished vascular circulation, nerve damage, immune system dysfunction, disrupted angiogenesis (Kolluru et al., 2012), and hyperglycemia (Baltzis et al., 2014). These factors substantially impair wound healing and tissue regeneration, increasing the likelihood of chronic infection. Tissue regeneration is a continuous process that progresses through four stages: hemostasis, inflammatory modulation, fibroblast proliferation, and tissue restoration (Trindade et al., 2024), and substantial delays in this process pose a grave clinical threat (Salama et al., 2026). Chronic ischemia and bacterial colonization transform these wounds into ulcerations called Diabetic Foot Ulcers (DFU). Methicillin-resistant *Staphylococcus aureus* (MRSA) is a prominent bacterial pathogen involved in a wide variety of infections, including dermal, tissue, septic, and bacterial infections, in diabetic foot ulcers (DFU) (Zafar et al., 2026). The World Health Organization (WHO) has listed MRSA as a “high priority” pathogen (Adekanmbi et al., 2023), thereby necessitating the discovery of new antimicrobials (Dighriri et al., 2025). Although MRSA is best known for its insusceptibility to β-lactam antimicrobials, many strains are multidrug-resistant (MDR) and therefore resist multiple antibiotic classes (Sharma et al., 2025). Moreover, chronic wound infections remain notoriously challenging to manage, wherein MRSA’s ability to form biofilm networks underscores its substantial role as a health hazard (Zafar et al., 2026). Biofilms are characterized by increased antimicrobial resistance, leading to failure of major antibiotics, thus prolonging MRSA infections (Kot et al., 2020). These biofilm-forming, multidrug-resistant microbes are identified as the main agents delaying tissue regeneration and increasing treatment failure rates in DFUs (Sultana et al., 2023). Biofilms are characterized by insusceptibility to host defense mechanisms, antibiotics, and stress conditions because the ECM provides layered protection (Singh et al., 2021). This, in turn, disrupts antimicrobial penetration, thereby altering metabolic homeostasis and reducing cell growth (Zafar et al., 2026). Advanced therapeutic approaches such as bacteriophage-based therapy, stimuli-responsive nanomaterials, and nanoparticles continue to evolve. Still, they are hindered by their inability to overcome microbial resistance, lack of specificity, and species-specific antimicrobial activity (Shi et al., 2026). Thus, emerging technologies such as nanotherapeutics are required to combat such chronic wound conditions. These pioneering strategies enhance their activity by selectively delivering them directly to the site of action to retain their bioavailability while having the ability to improve their safety of biological application, maintaining sustained diffusion of active substances over prolonged time, enhancing solubility of hydrophobic therapeutics, and enhancing antimicrobial diffusion into infections, while their small size is accountable for their enhanced permeation into tissue matrices (Zhao et al., 2025). One such emerging therapeutic agent-based carrier system gaining popularity is the nanovesicle, such as a microemulsion. Microemulsions are thermodynamically favored, homogeneous, optically clear dispersions of lipid and aqueous phases stabilized by an interfacial surfactant layer (Ebrahimi et al., 2026), with hydrodynamic diameters ranging from 10 to 200 nm (Sarhan et al., 2025). The term “microemulsions” was introduced in 1959 by Professor Jack H. Shulman of Columbia University. Microemulsions are characterized by reduced droplet diameter, thermostability, extended storage capability, reduced interfacial energy, negligible interfacial tension, simple formulation, transparency, large surface area, and enhanced dissolution (Lwin et al., 2026). The microemulsion system’s antimicrobial properties may stem from the oil’s inherent antibacterial activity and from encapsulating water within its structure, which limits microbial growth (Ghosh et al., 2026). The minuscule droplet size and negligible interfacial tension increase absorption and penetration of therapeutic agents, enhancing their utility as therapeutic carriers. Essential oils derived from aromatic plants, such as clove buds and cinnamon leaves or bark, are well recognized for their anti-inflammatory, ROS-scavenging, antimicrobial, and potential wound management properties (Asiri and Venkatesan, 2025), while comprising various bioactive compounds, including alkaloids, polyphenols, saponins, glycosidic compounds, and terpenoids (Osanloo et al., 2023). Eugenol (4-allyl-2-methoxyphenol), a hydroxyphenylpropene (Elwakil et al., 2026), is among the primary constituents of clove essential oil. It has gained popularity for its strong antimicrobial, anti-inflammatory, analgesic, and neuroprotective properties (Scalia et al., 2026), attributable to its free hydroxyl group, which can bind microbial endocrine modulators and disrupt their actions (Elwakil et al., 2026). Clove oil, a volatile aromatic compound derived from the buds of *Syzygium aromaticum*, exhibits intrinsic pesticidal, antibacterial, ROS-eliminating, and antiviral properties that disrupt membrane integrity in Gram-positive organisms, thereby altering gene expression via its Eugenol constituent (Luo et al., 2025). Cinnamon oil exhibits potent antibacterial and antifungal activities, attributed to its phenol-rich constituents (Kefayat et al., 2022). These bioactive constituents compromise microbial membrane integrity, leading to structural damage and subsequent ion leakage (Raeisi et al., 2015). Trans-cinnamaldehyde also exerts its antimicrobial activity by suppressing the bacterial enzyme acetyl-CoA carboxylase (Meades et al., 2010). The benefits of these essential oils are limited by their pronounced tendency of poor water solubility (Oun et al., 2022), evaporation and degradation under heat, light, or elevated temperatures. Moreover, drugs such as the glycopeptide antibiotic Vancomycin and the semisynthetic Methicillin, which are first-line treatments for MRSA and penicillinase-resistant infections, are becoming increasingly ineffective against MRSA. MRSA harbors the *mecA* gene, which confers resistance by encoding the modified Penicillin-Binding Protein (PBP2a) (Llarrull et al., 2009), thereby disrupting its ability to bind β-lactam antibiotics (Ambade et al., 2023), such as Methicillin, and hindering cell wall synthesis. Vancomycin exerts its antibacterial effects by binding to the D-Ala-D-Ala terminal residues of peptidoglycan precursors (Hughes et al., 2017), thereby inhibiting bacterial cell wall biosynthesis. Some MRSA strains develop thicker cell walls, limiting antibiotic entry, whereas in other strains the terminal binding sequence changes to Lac (Hu et al., 2016), leading to Vancomycin-Intermediate (VISA) and Vancomycin-resistant (VRSA) *Staphylococcus aureus* strains (Selim, 2022). Thus, developing carrier systems that encapsulate oils and bioactive compounds, such as antibiotics, for delivery to the potential wound site is crucial. The oil-in-water microemulsion system thus acts as an effective solution (Parın et al., 2023) for such cases, as it entraps the therapeutic constituents within the lipid matrix, thereby minimizing their plasma concentration while improving suitability for biological application (Kim and Park, 2026), and forming a protective barrier to prevent moisture or oxygen exchange between the wound bed and the surrounding environment (Sosa et al., 2025). Thus, this research aims to formulate and optimize a stable microemulsion system incorporating essential oils (Eugenol, Clove, and Cinnamon) and encapsulating active therapeutic agents (Vancomycin and Methicillin) to investigate their antibacterial and anti-inflammatory effects, along with potential to facilitate localized delivery to the site of action.

## 2 Materials and methods

### 2.1 Materials

Eugenol (Reagent Plus, 99%), Clove Oil (Reagent Plus, 99%), Cinnamon Leaf Oil (Reagent Plus, 99%), and non-ionic surfactant Tween 80 (polyethylene glycol sorbitan monooleate) were sourced from Sigma-Aldrich Chemicals Pvt. Ltd., Bengaluru, India. Mueller Hinton Broth (MHB), Mueller Hinton Agar (MHA), Brain Heart Infusion Agar (BHIA), and the antibiotics Methicillin Sodium Salt and Vancomycin Hydrochloride were purchased from HiMedia Laboratories Pvt. Ltd., Mumbai, India. 10× Phosphate Buffered Saline (PBS) was purchased from Sisco Research Laboratories (SRL) Pvt. Ltd., Mumbai, India. All studies were conducted using ultrapure water obtained from the Cascada BIO-water purifier (Pall India Pvt. Ltd., India) with resistivity greater than 18.3 mω cm^–1^.

### 2.2 Formulation of microemulsions

Initially, microemulsions were formulated using three essential oils, namely Eugenol, Clove, and Cinnamon leaf oil, along with the non-ionic surfactant Tween 80 to prepare an oil-in-water (O/W) microemulsion. For all microemulsions, the essential oil-to-surfactant ratio was set at 1:5 to assess clear system formation, with 1% v/v essential oil fixed for all 3 (EuME, CLME, CIME) based on a previous study (Gopikrishnan et al., 2026). After adding the appropriate oil and surfactant, the system was covered and stirred on a magnetic stirrer (SpinIt 4010; Tarsons Laboratory Equipment Pvt. Ltd., India) at 200 rpm for 5 min. The aqueous phase was then gradually added dropwise to the organic phase while stirring at 600 rpm under constant temperature conditions for 15 min to form a clear, homogeneous system. This homogeneous mixture was visually inspected during overnight storage at ambient temperature, away from light to observe changes such as flocculation, phase separation, or turbidity; wherein the absence of these indicates the formation of a stable microemulsion.

### 2.3 Formulation of drug-loaded microemulsion

Antibiotic stock solutions were prepared using the hydrophilic drugs – Vancomycin hydrochloride and Methicillin sodium salt through dissolution of 10 mg in 4 mL of sterile ultrapure water, vortexing for 10 min, and storing at 2 °C until use. This formulation was added to the above-mentioned organic phase (oil: surfactant) ratios of 1:5 by replacing the amount of deionized water from this mixture with this antibiotic solution, with the final proportion being (1:5:4) while maintaining its viscosity under similar stirring conditions to form Eug-Van, Clo-Van, Cin-Van; Eug-Met, Clo-Met, Cin-Met representing the different drug-loaded (vancomycin and methicillin) counterparts of Eugenol, clove, and cinnamon microemulsions, respectively. Visual observations were noted, and the samples were left overnight at ambient temperature, away from light, to monitor changes in their homogeneity.

### 2.4 Characterization studies

#### 2.4.1 Droplet size, polydispersity index (PDI), and zeta potential

The hydrodynamic diameter and polydispersity index (PDI) of the microemulsions and their drug-loaded counterparts were analyzed through dynamic light scattering (DLS) utilizing the Horiba Scientific Nanoparticle Analyzer (SZ-100, Japan) at ambient temperature conditions, where the beam of monochromatic light passing through the sample gets scattered due to the particles in Brownian motion. The scattering intensity determines the size of suspended particles. All samples were diluted at 1:3 with deionized water (Gopikrishnan et al., 2026) to minimize multiple-scattering effects. The electrophoretic velocity of the samples was investigated utilizing the 90 Plus Particle Size Analyzer (Brookhaven Instruments Corp., USA) to determine their surface charge and colloidal stability, wherein, under the application of an electric field, the velocity of the movement of the particles toward oppositely charged electrodes is correlated to their zeta potential by virtue of the Smoluchowski equation. All measurements were carried out at ambient temperature, and data were analyzed using Horiba’s software.

#### 2.4.2 Stability of microemulsions

The stability of the formulated microemulsions under thermal stress was evaluated through three cycles of freezing to −20 °C followed by thawing at room temperature (Long et al., 2024), with subsequent cycles of heating and cooling at 45 °C and 4 °C for 48 h, respectively. The kinetic Stability of the microemulsions was monitored with slight modifications by centrifuging the formulations at 1500 × *g* for 30 min (Haq et al., 2023). Formulations free of phase separation, flocculation, or turbidity were identified as optimal and selected for further characterization. Storage longevity was assessed by periodically monitoring globule diameter and polydispersity index over 3 months, with all experiments performed in triplicate.

### 2.5 Physicochemical attributes

#### 2.5.1 pH

Further characterization was performed only for the optimal formulations by measuring pH with a pH meter (LABMAN LMPH-10) to assess their ability to maintain normal skin conditions, stability, and storage efficiency. The measurement followed the steps: washing the probe with Milli-Q water, blotting dry, calibrating with the pH standard, rinsing it clean, and dipping it into the samples with gentle stirring until a stable reading appeared.

#### 2.5.2 Fourier transform infrared (FTIR) spectroscopy of emulsions

The microemulsions and their corresponding drug-loaded variants were scrutinized by Fourier transform infrared (FTIR) spectroscopy on a JASCO FT/IR-6800 (Jasco, India) at a scan rate of 2 cm^–1^ to assess the presence of the corresponding functional groups. For analysis, minuscule drops of Microemulsion were used, with an absorbance spectral range of 400–4000 cm^–1^, where infrared radiation absorbed by different functional groups at their corresponding frequencies and characteristic wavelengths, help confirm the presence of these functional groups and other associated interactions or modifications.

#### 2.5.3 Morphological assessment of microemulsion

The uniformity or distribution of the colloidal system, the primary particle diameter, and structure were analyzed using High-Resolution Transmission Electron Microscopy (HR-TEM), in which an electron beam passes through the sample and is further focused to produce a magnified image of the droplet size and shape. For sample preparation, the emulsions were diluted with Milli-Q water, subjected to water-bath sonication, and a minuscule drop of the emulsion was drop-cast onto a copper grid and air-dried under constant-temperature conditions. The TEM images were further acquired using an FEI Tecnai G2 F20 X-TWIN (accelerating voltage: 200 kV) at room temperature.

### 2.6 Antimicrobial studies

#### 2.6.1 Antimicrobial susceptibility testing (AST)

Bacterial cultures MRSA-CI 1 and MRSA-CI 2, provided by the Clinical Microbiology Department of Christian Medical College, were supplied as Methicillin-Resistant *Staphylococcus aureus* isolates based on antimicrobial and molecular characterization of their resistance profiles performed by the source laboratory. In contrast, MTCC 96 served as a reference strain to qualitatively compare the efficacy of drug-loaded and plain emulsions with free drug solutions, essential oils, and vehicle controls. Initially, MHA plates were freshly prepared by dispensing agar solutions onto culture plates and allowed to solidify. Next, adjusted bacterial cultures were used to create a bacterial lawn on these plates using sterile cotton swabs. Each strain was divided into three plates – one for each Microemulsion and a well diffusion approach was used wherein wells were created on these plates with pipette tips, and 100 μl of these emulsions were poured into such wells situated on thirds of each plate – one for microemulsion alone and the other two halves for their respective drug-loaded counterparts. These plates were covered and allowed to diffuse by incubation for 24 h at 37 °C to determine the inhibition zones.

#### 2.6.2 Evaluation of minimum inhibitory concentration (MIC)

Comparative analysis of the antibacterial efficacy of the different microemulsions (EuME, CLME, CIME) and their drug-loaded counterparts (Eug-Van, Clo-Van, Cin-Van; Eug-Met, Clo-Met, Cin-Met) was determined against Methicillin-resistant *Staphylococcus aureus* (Gram-positive pathogen) strains of MRSA-CI 1 and MRSA-CI 2 and the reference strain MTCC 96. The broth microdilution method was used as per CLSI guidelines (CLSI M07), and the main stock solutions were diluted 1:1 to prepare aliquots. The operational solutions were mixed with the aforementioned overnight bacterial solutions and adjusted to 0.05 OD_600_ (Thanakarn et al., 2026) (approximately 1 × 10^7^–5 × 10^7^ CFU/mL) in a 96-well plate. MIC, or Minimum Inhibitory Concentration, is the lowest concentration of an antibacterial compound that suppresses noticeable microbial growth after 12–24 h of incubation at 37 °C. It is mainly used to confirm resistance and determine breakpoints. MHB media alone served as the negative control (blank), without bacterial suspension. In contrast, MHB with bacterial suspension served as the positive (growth) control, with plain oils as the active controls and surfactant Tween 80 as the vehicle control. The free drug solutions and drug-loaded emulsions were in the range of 64 μg/mL–0.03125 μg/mL, whereas the plain emulsions and free essential oils were in the range of 20 μL/mL–0.00976 μL/mL of the active essential oil in the emulsion, and the vehicle control was in the range of 100 μL/mL–0.0488 μL/mL of the surfactant in the emulsion. This 96-well microtiter plate was maintained at 37 °C overnight to determine MIC values, with the last well’s concentration serving as the final reading before turbidity was observed using the Turbidometry assay (Haq et al., 2023).

#### 2.6.3 Biofilm screening study

The ability to form biofilms for these strains was evaluated through a biofilm screening assay with slight modifications to a study by Ragupathi et al. (2023), wherein 200 μL of overnight culture was adjusted to OD_625_ for inoculation in a 96-well plate and incubated at 37 °C for 48 h to allow the bacteria to establish the biofilms. In contrast, wells containing only media served as the negative control. After the specified duration, the biofilms were rinsed with sterile water, followed by staining with 200 μL of 0.1% (w/v) Crystal Violet for 15 min, washed with sterile water, followed by destaining with 200 μL of 33% (v/v) glacial acetic acid for 5 min at room temperature before being subjected to absorbance measurement in a microplate reader at OD_570nm_. The assay was performed in triplicate for all strains, and the cutoff OD (ODc) was calculated as the mean OD of the negative control plus three standard deviations. Accordingly, they were classified depending upon the criterion: OD < ODc = poor biofilm formers; ODc < OD ≤ 2 × ODc = weak biofilm formers; 2 × ODc < OD < 4 × ODc = moderate biofilm formers; and OD ≥ 4 × ODc = strong biofilm formers.

#### 2.6.4 Evaluation of biomass-reduction endpoint

We evaluated the potential of the optimized drug-loaded test formulations to reduce biofilm biomass of clinical isolates and the reference strain in accordance with (Franklyne et al., 2019), with slight modifications. Initially, overnight cultures were adjusted to OD_625_, and 200 μL of the adjusted culture was inoculated into a 96-well plate and incubated for 48 h at 37 °C. After the specified time point, the cultures were removed, and the wells were washed with sterile water to remove planktonic cells; then 200 μL of test solutions or drug-loaded microemulsions were added to the treated samples. In contrast, the media with culture acted as the growth control for 24 h. The test solutions were carefully removed from the wells, rinsed with sterile water, and air dried followed by staining with 0.1% (w/v) Crystal Violet for 15 min, rinsing, air drying and destaining with 200 μL of 33% (v/v) glacial acetic acid at room temperature for measurement via optical inference wherein all the results were validated upon the concentrations compared alongside the MIC values.

#### 2.6.5 Evaluation of antimicrobial synergy testing

Antimicrobial efficacy of the emulsions and drug solutions in combination was determined using the checkerboard assay, following (El-Demerdash et al., 2023), with slight modifications. Briefly, we prepared serial dilutions of the antibiotics and plain emulsions at concentrations four times above the MIC value. We diluted the stock solutions along the ordinate and abscissa, respectively, in a 96-well plate so each well contained 50 μL of each compound. We then added 100 μL of overnight culture adjusted to OD_625_ and incubated it at 37 °C for 18–24 h. Readings were recorded by observing turbidity in the respective wells, and interaction attributes were analyzed by calculating the Fractional Inhibitory Concentration (FIC) indices. The individual FIC of the drugs and emulsions were analyzed by dividing their MIC in combination by their MIC alone and the summation of both FICs (FICI = FIC_*antibiotic*_ + FIC_*emulsion*_) was used to determine whether their activity was synergistic (FICI ≤ 0.5; enhanced activity in combination compared to activity alone), additive (0.5 < FICI ≤ 1; combined activity is the sum of the individual agents expected outcomes), indifferent (1 < FICI ≤ 4; no considerable increase in the inhibitory activity) or antagonistic (FICI > 4; reduces the activity and efficacy of the components) (Lorian, 2005).

#### 2.6.6 Evaluation of time-kill kinetics

Killing kinetics upon exposure to drug-loaded microemulsions were determined using the time-kill assay, following (Yazgan, 2022) with some modifications. Briefly, 100 μL of the test solutions were added to a 96-well plate at 2 × MIC, followed by an equal volume of overnight bacterial culture adjusted to OD_625_, and incubated at 37 °C for 24 h; the broth with culture served as the growth control. At the 0th, 2nd, 4th, 6th, 8th, and 24th hour, 20 μL of these solutions were serially diluted with 180 μL of sterile water, and 10 μL of these dilutions were spotted onto sterile MHA plates, which were then incubated at 37 °C overnight to calculate viable colony counts.

### 2.7 Evaluation of encapsulation efficiency

The efficiency of encapsulation for the drug-loaded microemulsions was evaluated utilizing UV-visible spectrophotometry in accordance to a study by Jadhav et al. (2023), wherein known volume of the drug loaded microemulsion was diluted with the specific solvent, centrifuged and supernatant was subjected to spectrophotometric analysis in a UV spectrophotometer (Jenway 6850) at the predetermined λ_*max*_ values for the respective drugs Vancomycin and Methicillin at 280 nm and 330 nm, wherein the drug concentrations were interpolated from their respective standard calibration curves. The entrapment efficiency was calculated using the following equation:


$$\%EE=\left(\frac{\begin{array}{c} A\,m\,o\,u\,n\,t\,o\,f\,d\,r\,u\,g\,e\,n\,t\,r\,a\,p\,p\,e\,d \end{array}}{A\,m\,o\,u\,n\,t\,o\,f\,d\,r\,u\,g\,t\,a\,k\,e\,n}\right)*100$$

### 2.8 Drug release profiles

To determine the release behavior of Vancomycin and Methicillin from the drug-loaded microemulsions, pH 7.4 was selected in accordance with earlier studies (Miranda-Calderon et al., 2022), as it corresponds to the initial inflammatory phase or acute wound conditions, to assess drug release under mimicked wound pH conditions. Freshly prepared 1× Phosphate Buffered Saline (PBS) made from a 10× stock solution was used as the release medium because it is biocompatible, maintains a stable pH through its buffering capacity, provides ionic strength, and supports sink conditions, all of which mimic the *in vivo* environment. The pH was adjusted with HCl and NaOH and stored in the refrigerator until use. We used the beaker method with dialysis membranes (Dialysis Membrane – 60, MWCO – 12–14 kDa, HiMedia Laboratories) cut into 1 × 1 cm squares. A fixed volume of 1 mL of drug-loaded microemulsions was loaded into each square, secured on both sides with threads, and suspended in beakers filled with release medium to a total volume of 50 mL. All setups were maintained at room temperature with uniform stirring at 150 rpm. At predetermined time points, fixed aliquot volumes of 1.5 mL each were retrieved from these beakers and replenished with an equal volume of fresh buffer every 1 h until the 12th hour and at the 24th hour to maintain sink conditions. The absorbance of these aliquots was measured using a UV spectrophotometer at the respective λ_*max*_ values of 280 nm and 330 nm for Vancomycin and Methicillin, respectively, to extrapolate concentration values from the respective standard calibration curves and evaluate the cumulative percentage drug release. The experiment was performed in triplicate, and subsequent readings were evaluated for statistical significance.

### 2.9 Statistical analysis

Experimental studies and their corresponding quantitative data were analyzed, with results for independent biological replicates being expressed as Mean ± SD (Standard Deviation) for stability study, biofilm screening assay and release kinetics whereas for experiments such as size, pH, TEM morphology, FTIR, entrapment efficiency, antimicrobial assays like MIC, AST, Antibiofilm, Checkerboard and Time kill assays where there was no biological replicates are mentioned descriptively wherein all the graphs for the associated results were prepared using GraphPad Prism 10 and Origin 2026b, whereas images were analyzed using ImageJ.

## 3 Results

### 3.1 Preparation of the microemulsion conformity

This study investigates microemulsions containing Eugenol, Clove, and Cinnamon oils, with these essential oils as the organic lipid phase and Tween 80 as the non-ionic surfactant that emulsifies and stabilizes the system, using ultrapure Milli-Q water as the aqueous phase. All three microemulsion systems utilized optimal ratios of 1:5:4 for essential oils (Eug/Clo/Cin): surfactant (Tween 80): water (Milli-Q) constituents, resulting in an optically clear, translucent formulation upon preparation, which, upon overnight storage in dark conditions, showed complete absence of phase separation, creaming, flocculation, or turbidity (Figure 1), confirming their physical stability for use in further physicochemical characterizations.

**FIGURE 1:**
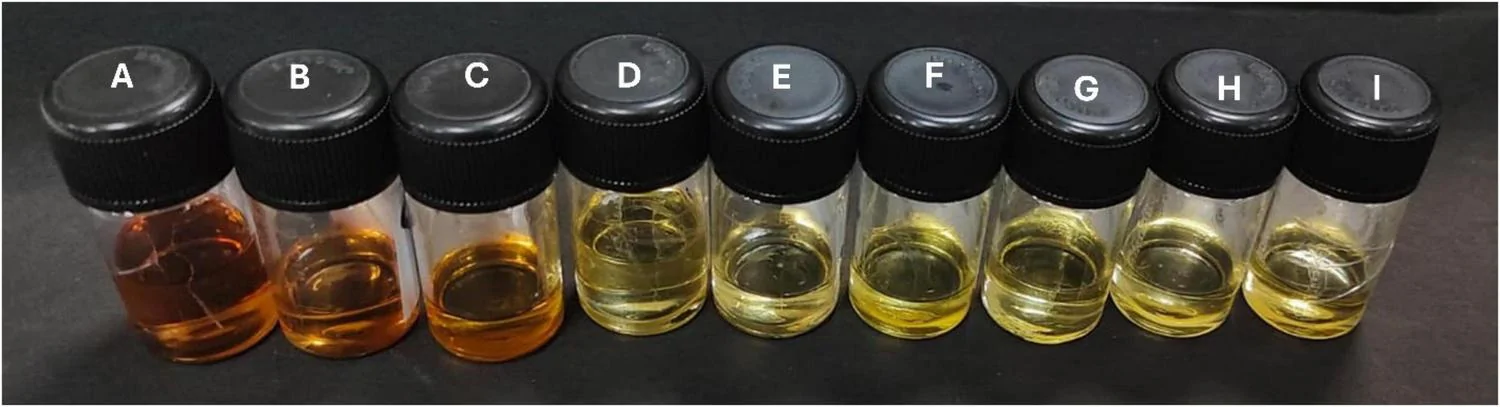
Optically translucent microemulsions of eugenol, clove, and cinnamon oils in order: **(A)** EuME, **(B)** Eug-Van, **(C)** Eug-Met, **(D)** CLME, **(E)** Clo-Van, **(F)** Clo-Met, **(G)** CIME, **(H)** Cin-Van, **(I)** Cin-Met.

### 3.2 Formulation of the drug-encapsulated microemulsion systems

Blank emulsion formulations served as controls. In contrast, the drug-loaded counterparts were prepared by slightly modifying the plain microemulsions by adding the aqueous component. Vancomycin and Methicillin were incorporated into the respective formulations using three different oil systems (Eugenol, Clove, and Cinnamon essential oils). Both of these therapeutic agents are hydrophilic and thus, their stock solutions were prepared in deionized water and replaced with the aqueous phase of the plain emulsion system for thorough solubilization and incorporation of these drugs into the droplets, to yield the different drug-loaded counterparts of the above essential oil emulsions for Vancomycin (Eug-Van, Clo-Van, Cin-Van) and Methicillin (Eug-Met, Clo-Met, and Cin-Met), respectively. These formulations were stored under the same physiological conditions as the control formulations and showed optimal physical stability, with no flocculation or turbidity, confirming their suitability for further characterization (Figure 1).

### 3.3 Characterization studies: size, PDI, and zeta potential

We investigated all microemulsions for average hydrodynamic diameter and polydispersity index using a nanoparticle analyzer. The average droplet diameter and Polydispersity index for all emulsion systems were within the optimal and acceptable ranges of 10–50 nm and a PDI less than 0.5 (Table 1). The average effective diameters (Z_*avg*_) for the Eugenol, Clove, and Cinnamon oil microemulsions were 26.5 nm, 31.8 nm, and 17.7 nm, respectively, with their PDI values being 0.344, 0.322, and 0.434, respectively. For the Vancomycin-loaded formulations (Eug-Van, Clo-Van, Cin-Van), the respective droplet sizes were less than 20 nm (17.1 nm, 15.7 nm, and 18.0 nm), and the PDI was 0.365, 0.313, and 0.379, respectively. Similarly, for the Methicillin-incorporated formulations, the diameters were 22.9 nm, 16.9 nm, and 15.8 nm, with dispersion index measurements of 0.380, 0.340, and 0.308, respectively (Figure 2). All values for the effective diameter and polydispersity index indicate a uniform distribution of the dispersed particles, augmented kinetic stability, and increased interfacial area, which may enhance permeation and interaction with biological membranes. The determination of the zeta potential of the emulsion systems further confirms the colloidal Stability of the systems. Surface charge density determines the likelihood of coalescence or Ostwald ripening; strong charges promote repulsion between adjacent molecules, preventing aggregation. The zeta potential measurements for all the formulations exhibited negative surface charges in the optimal realm of ±30 mV (Table 1), with their values being −9.04 mV, −17.88 mV, −19.40 mV for control formulations (EuME, CLME, CIME), −11.15 mV, −8.08 mV, −6.71 mV (Eug-Van, Clo-Van, Cin-Van) for Vancomycin, and −9.80 mV, −9.02 mV, −9.86 mV (Eug-Met, Clo-Met, Cin-Met) for Methicillin-loaded counterparts, respectively (Figure 3). These values indicate that the negative charges on the particles produce modest electrostatic repulsion among the droplets and thus might not account for the predominant stabilizing mechanisms. The presence of the non-ionic surfactant could be responsible for the steric hindrance, thereby preventing particle aggregation or coalescence and contributing to the formation of a relatively stable emulsion system.

**TABLE 1:** Physicochemical characterizations of the optimized microemulsions for their average droplet size, zeta potential, and pH values.

S. No.	Sample (emulsions)	Z average (nm)	PDI	Zeta potential (mV)	Mobility (μ/s)/(V/cm)	pH
1.	Eugenol	26.5	0.344	−9.04	−0.71	5.14
2.	Vancomycin-Eugenol	17.1	0.365	−11.15	−0.87	5.62
3.	Methicillin-Eugenol	22.9	0.380	−9.80	−0.77	5.92
4.	Clove	31.8	0.322	−17.88	−1.40	5.21
5.	Vancomycin-clove	15.7	0.313	−8.08	−0.63	5.65
6.	Methicillin-clove	16.9	0.340	−9.02	−0.70	5.87
7.	Cinnamon	17.7	0.434	−19.40	−1.52	5.4
8.	Vancomycin-cinnamon	18.0	0.379	−6.71	−0.52	5.94
9.	Methicillin-cinnamon	15.8	0.308	−9.86	−0.77	6.14

**FIGURE 2:**
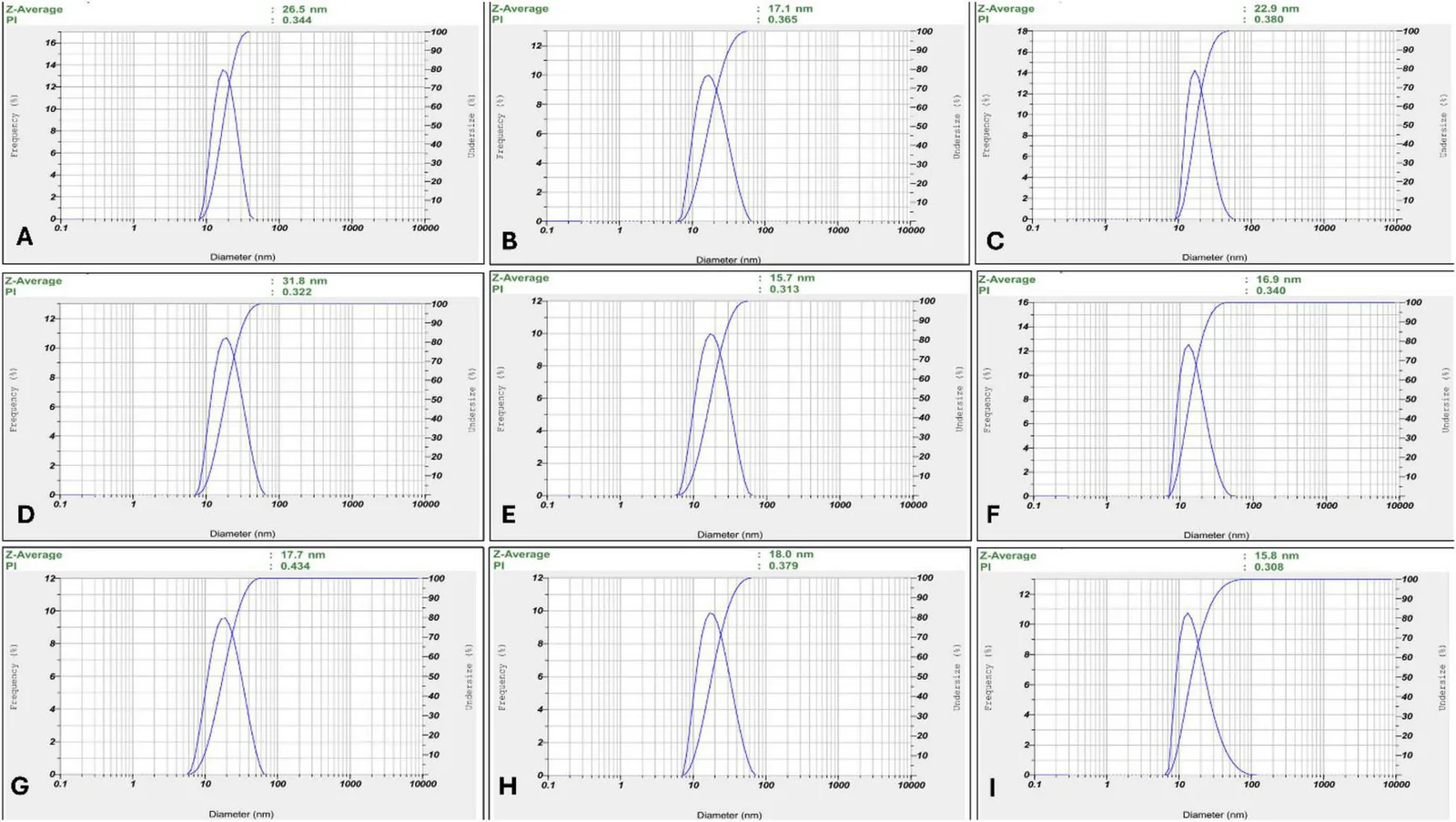
Hydrodynamic diameter and polydispersity index (PDI) of the microemulsions: **(A)** EuME, **(B)** Eug-Van, **(C)** Eug-Met, **(D)** CLME, **(E)** Clo-Van, **(F)** Clo-Met, **(G)** CIME, **(H)** Cin-Van, **(I)** Cin-Met.

**FIGURE 3:**
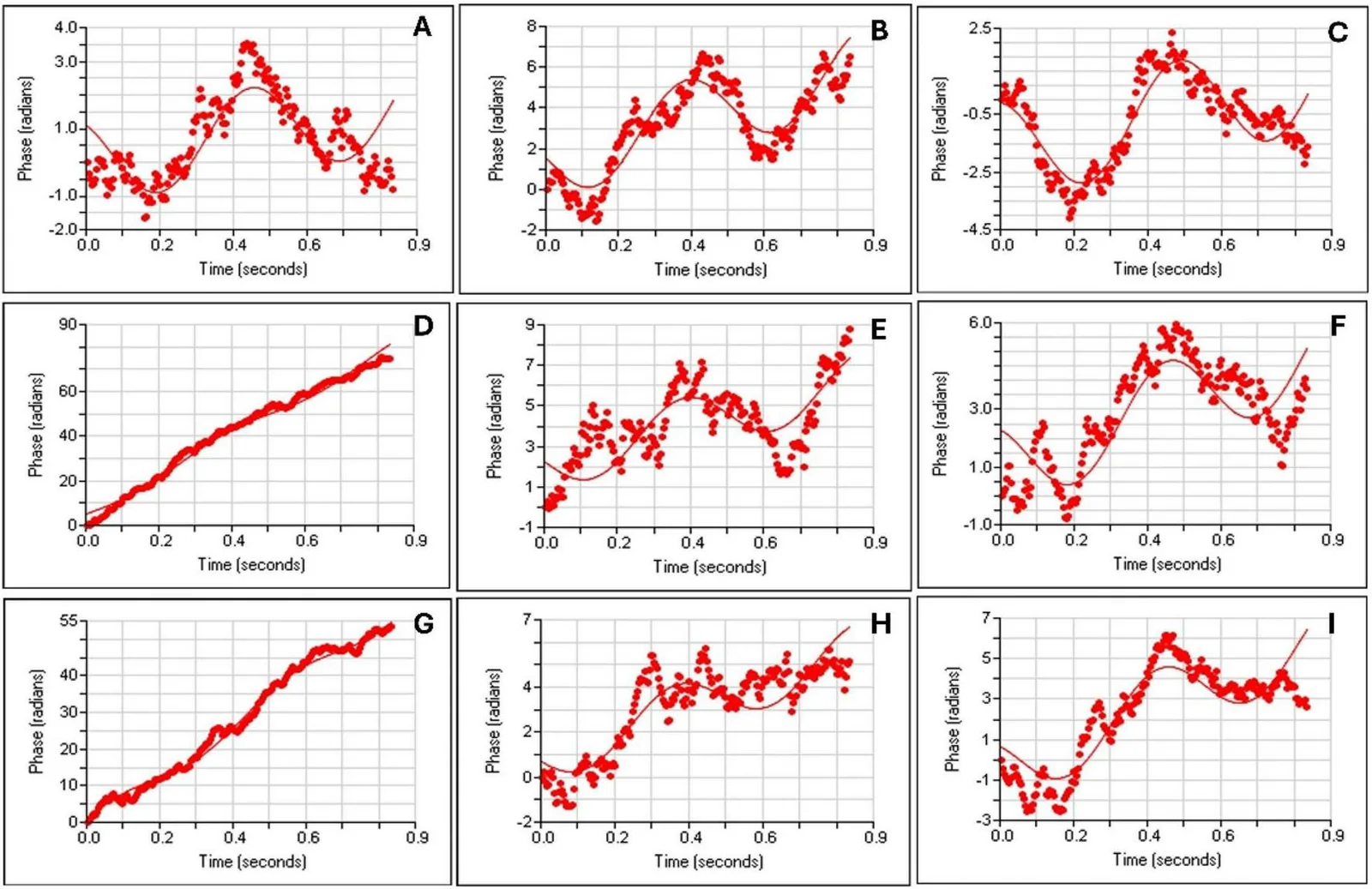
Zeta potential of the optimized microemulsions: **(A)** EuME, **(B)** Eug-Van, **(C)** Eug-Met, **(D)** CLME, **(E)** Clo-Van, **(F)** Clo-Met, **(G)** CIME, **(H)** Cin-Van, **(I)** Cin-Met.

### 3.4 Stability of emulsions

The equivalent thermodynamic stability of microemulsions was evaluated through consecutive freezing-thawing and heating-cooling cycles after centrifugation. Colloidal stability for the formulated microemulsions was further investigated by storing them at room temperature in the dark for about 3 months to assess any changes in droplet size or optical Stability. All 9 formulations, including the control and drug-loaded ones, showed optimal hydrodynamic diameter and distribution index (Figures 4A,B), without any stratification, flocculation, or turbidity, well within the range of stable emulsion systems, with effective diameters within 30 nm and Polydispersity Index within an acceptable range of less than 0.5, indicating a stable system, with uniformly distributed droplets that render good stability, lesser aggregation chances, good reproducibility, and preservation of homogeneity.

**FIGURE 4:**
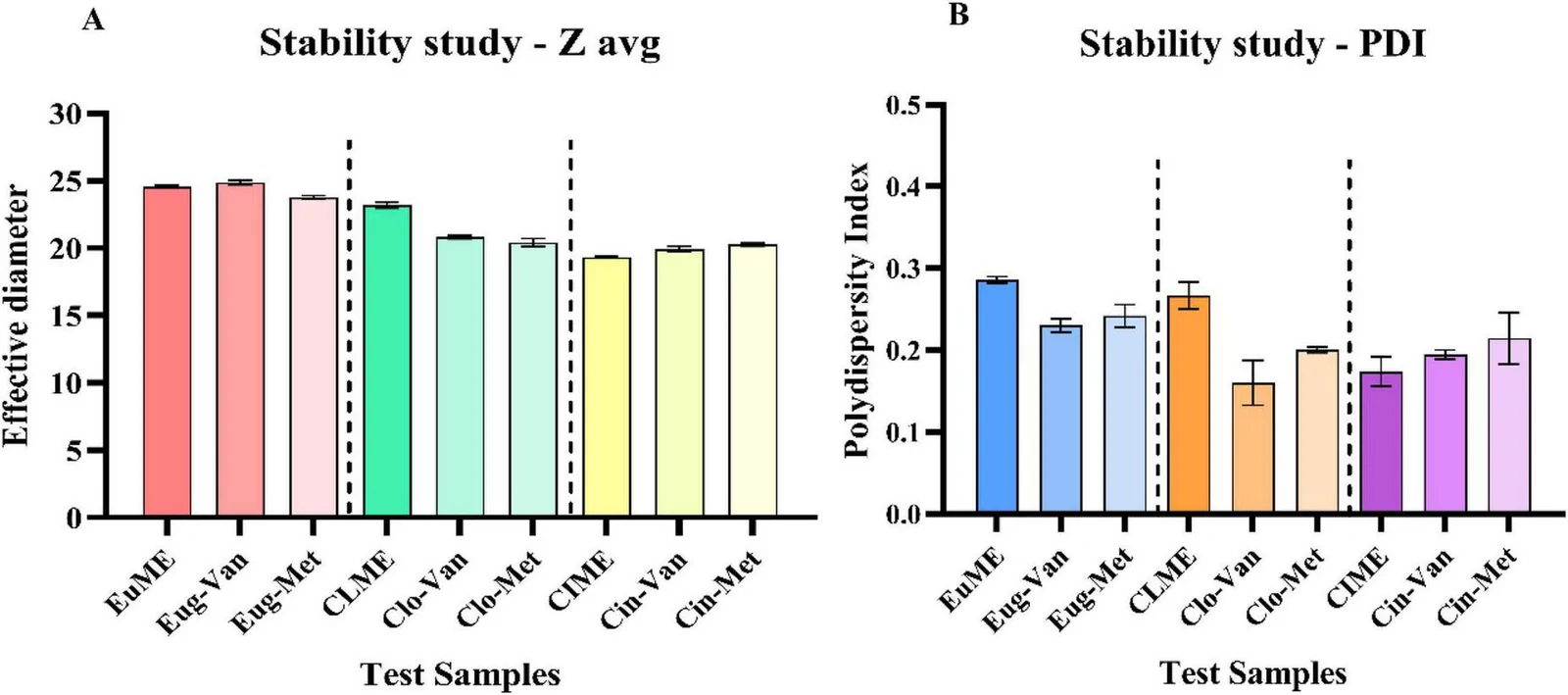
Stability study results for all the microemulsions, with **(A)** showing the effective hydrodynamic diameter and **(B)** showing the polydispersity index (PDI) for the same within the optimal range; values are represented as Mean ± SD, *n* = 3.

### 3.5 pH of emulsions

All formulations were tested for pH, a parameter used to assess stability. The pristine emulsions displayed pH values of 5.14, 5.21, and 5.4 for Eugenol, Clove, and Cinnamon microemulsions, respectively. For those comprising the Vancomycin moieties, the values were 5.62, 5.65, and 5.94 for Eug-Van, Clo-Van, and Cin-Van emulsions, respectively. Accordingly, for the Methicillin-loaded counterparts, the exhibited values were 5.92, 5.87, and 6.14, respectively, for the Eug-Met, Clo-Met, and Cin-Met microemulsions (Table 1). All formulations exhibited acidic pH, which is crucial for potential healing because it mimics natural skin pH, reverses the alkaline environment of the chronic wound bed, inhibits pathogen growth, increases oxygen perfusion, and accelerates tissue regeneration to improve wound healing, which could be useful in future wound management applications.

### 3.6 FTIR analysis

The FTIR spectra of Eugenol, Eug-Van, and Eug-Met; Clove, Clo-Van, and Clo-Met; and Cinnamon, Cin-Van, and Cin-Met exhibited prominent peaks at different wavenumbers corresponding to their respective functional groups. The wavenumbers at 1457 cm^–1^, 2923 cm^–1^, and 2857 cm^–1^ corresponded to C-H scissoring of Tween 80, along with its symmetric and asymmetric modes. The wavenumbers around 1248 cm^–1^ and 1033 cm^–1^ depict the antisymmetric C-O-C and C-N stretching modes of the primary amine group in Vancomycin, while those around 1740 cm^–1^ and 1630 cm^–1^ correspond to C = O stretching vibration of the β-lactam moiety and the Amide I band, and to the asymmetric stretching vibration of the carboxylate moiety of Methicillin sodium salt. For Eugenol, peaks were observed at around 1515 cm^–1^, 1267 cm^–1^, 994 cm^–1^, 1092 cm^–1^, and 1348 cm^–1^, indicating aromatic C = C stretching vibrations, out-of-plane bending vibrations, C-O stretching vibrations, and C-H bending, respectively. For Clove, wavenumbers at 914 cm^–1^, 992 cm^–1^, 1269 cm^–1^, 1084 cm^–1^, and 1515 cm^–1^ were assigned to =C-H aliphatic bending, C-O-C asymmetric stretching, C-O stretching, and aromatic C = C vibrations, respectively, due to Eugenol, the primary component of clove oil. In both cases, peaks around 1600 cm^–1^ and 950 cm^–1^ indicated C = C stretching and out-of-plane bending (950 cm^–1^ = C-H) of the allyl groups. In case of Cinnamon, peaks centered around 1500 cm^–1^, 1630 cm^–1^, 1345 cm^–1^, 1270 cm^–1^, 1085 cm^–1^, 946 cm^–1^, 910 cm^–1^ and 792 cm^–1^ depicted CH_*x*_ deformation vibration, C = O aldehyde stretching, -C-H (methyl) bending, C-O stretching of the Ar-OH group, C-O-C linkage in aromatic acid esters, aromatic ring substitutions, CH bending (RCH = CH2) and out of plane stretching for the 1,2,4 – trisubstituted aromatic ring, respectively (Figure 5). Infrared Spectra of the plain microemulsions and their drug-encapsulated modifications show no prominent differences in peak positions, with only minor shifts and broadening in the drug-loaded samples due to intermolecular interactions, indicating optimal compatibility of the modifications and successful drug incorporation.

**FIGURE 5:**
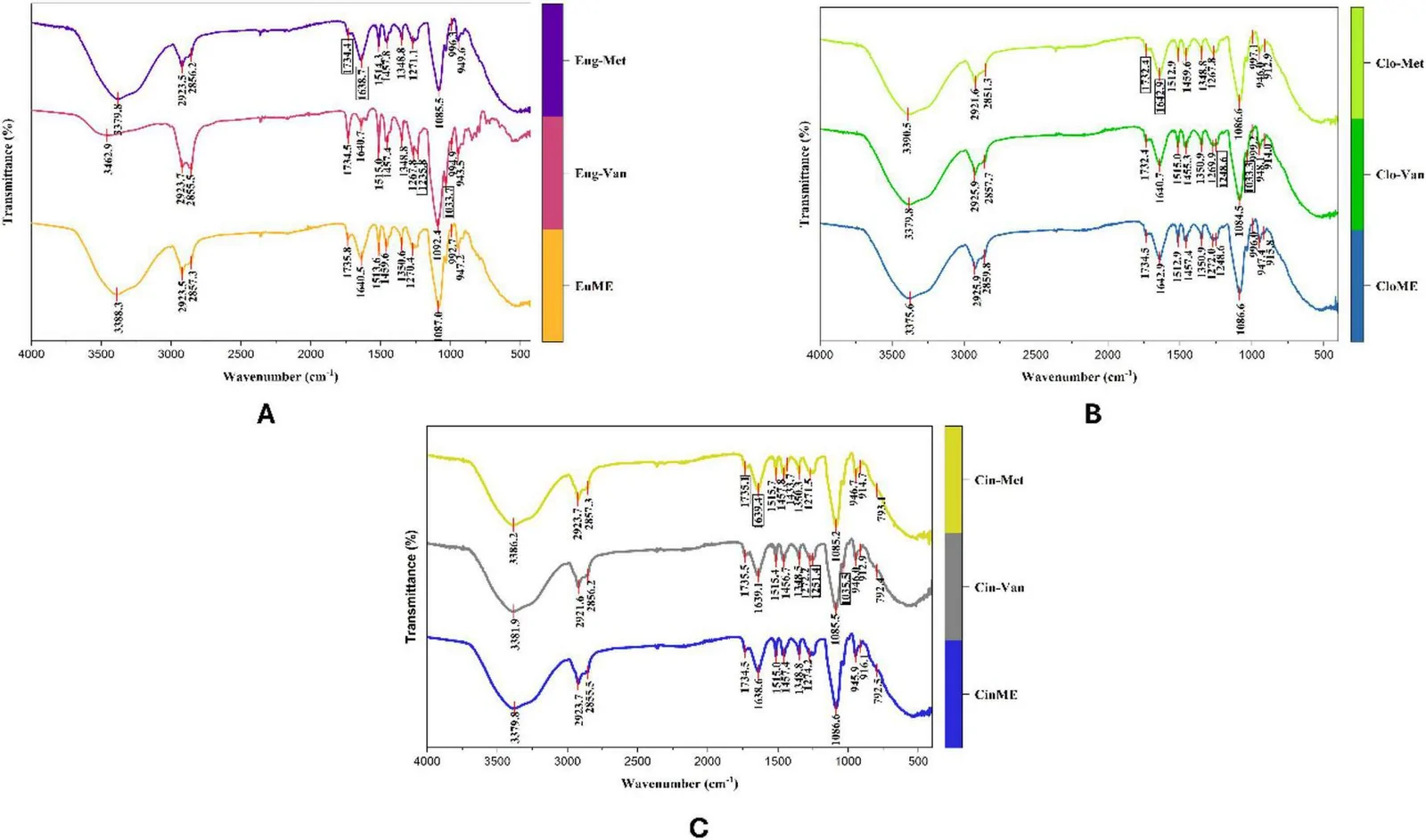
FTIR Spectra of the plain microemulsions in comparison with their drug-loaded modifications in order of panel **(A)** EuME, Eug-Van and Eug-Met, **(B)** CLME, Clo-Van and Clo-Met, **(C)** CIME, Cin-Van and Cin-Met.

### 3.7 TEM assessment

Analysis of TEM micrographs revealed the morphology and structural parameters of the emulsion droplets. The plain EuME, CLME, and CIME emulsions exhibited values of 24.36 nm, 29.04 nm, and 92.57 nm, respectively. The Eug-Van, Clo-Van, and Cin-Van emulsions demonstrated average diameters of 15.76 nm, 7.46 nm, and 19.28 nm, respectively. Accordingly, the Eug-Met, Clo-Met, and Cin-Met emulsions displayed values of 18.63 nm, 26.36 nm, and 15.86 nm, respectively. Most formulations displayed spherical-oval droplet morphology (Figure 6), indicating a stable emulsion, uniform distribution, and a smoother interfacial area, as confirmed visually.

**FIGURE 6:**
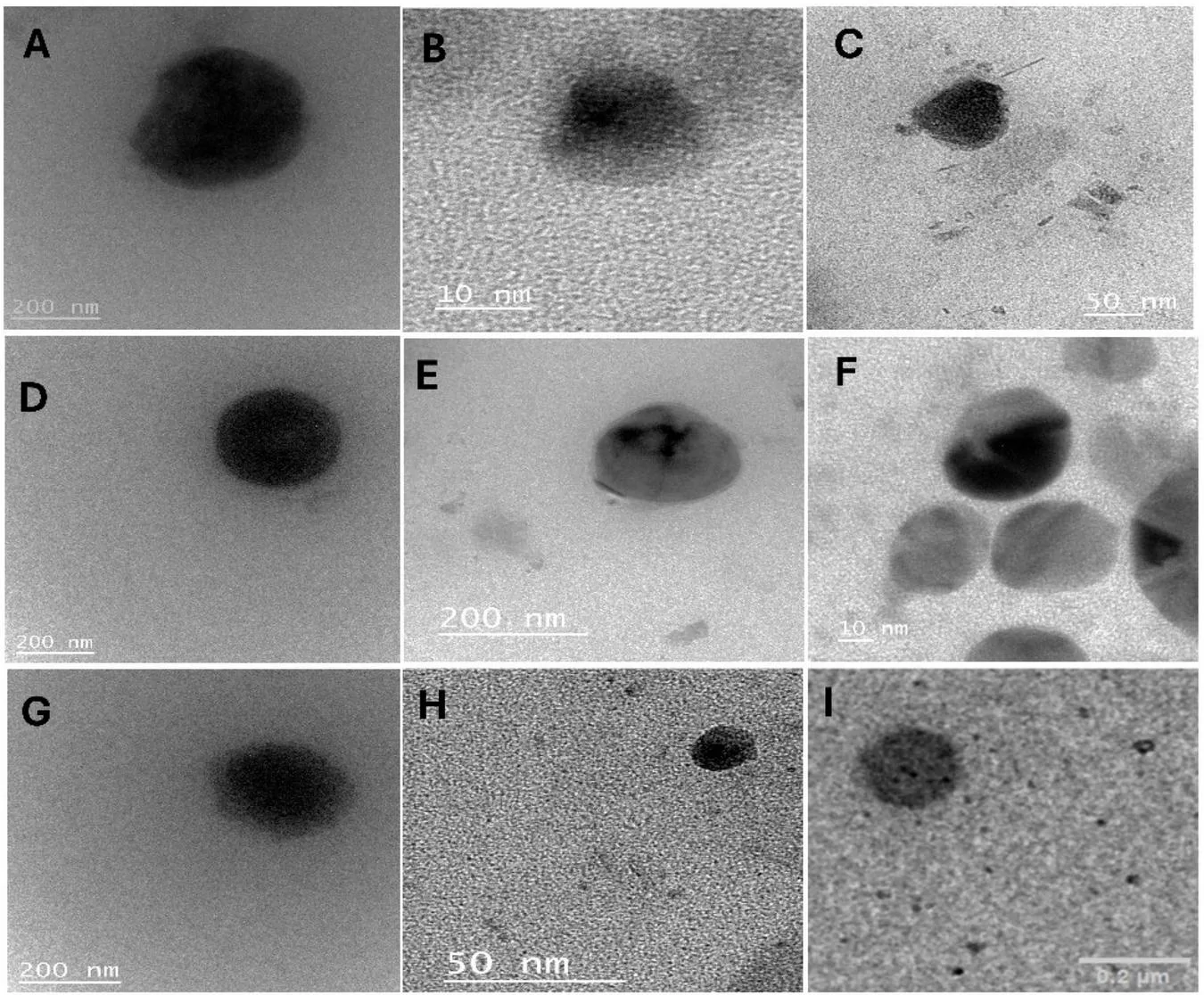
TEM micrographs exhibiting spherical to oval morphology for all the microemulsions: **(A)** EuME, **(B)** Eug-Van, **(C)** Eug-Met, **(D)** CLME, **(E)** Clo-Van, **(F)** Clo-Met, **(G)** CIME, **(H)** Cin-Van, **(I)** Cin-Met.

### 3.8 Antibacterial studies

#### 3.8.1 Well diffusion assay

Antibacterial susceptibility testing displayed prominent zones of inhibition against both the MRSA strains and the reference strain. For MRSA-CI 1, plain Eugenol, Eugenol vancomycin, and Eugenol methicillin displayed diameters of 8 mm, 18 mm, and 14 mm, respectively. In contrast, for MRSA-CI 2, the zone diameters were 10 mm, 14 mm, 12 mm, and 12 mm, 18 mm, and 18 mm for MTCC 96, respectively. Similarly, for clove, the plain emulsion, clove vancomycin, and clove methicillin formulations exhibited diameters of 6 mm, 16 mm, and 14 mm for MRSA-CI 1; 6 mm, 16 mm, and 12 mm for MRSA-CI 2; and 11 mm, 18 mm, and 17 mm for MTCC 96, respectively. Accordingly, cinnamon emulsion, along with its Vancomycin- and Methicillin-loaded counterparts, showed inhibition zone diameters of 4 mm, 12 mm, and 10 mm against MRSA-CI 1 and 4 mm, 14 mm, and 10 mm against MRSA-CI 2, whereas 12 mm, 17 mm, and 17 mm against MTCC 96, respectively. For the pristine drug solutions, the inhibition zone diameters were 18 mm, 14 mm and 18 mm for Vancomycin, and 14 mm, 10 mm and 19 mm for Methicillin, for MRSA-CI 1, MRSA-CI 2 and MTCC 96, respectively whereas the diameters for free Eugenol, Clove, Cinnamon oil and Tween 80 were 20 mm, 18 mm, 14 mm, 14 mm; 20 mm, 17 mm, 17 mm, 14 mm and 19 mm, 17 mm, 17 mm, 14 mm for MRSA-CI 1, 2 and MTCC 96, respectively (Figure 7). The plain emulsions, although displaying minimal zones due to their inherent antimicrobial activity, showed more prominent zones of inhibition. In the case of Vancomycin-loaded formulations, Eug-Van and Clo-Van had better antibacterial efficacy compared to other drug-loaded formulations against MRSA-CI 1 and 2, respectively, under the given conditions, whereas for Methicillin-loaded moieties, Clo-Met had the most prominent activity against both strains, indicating comparatively measurable formulation-dependent antimicrobial activity of the drugs by encapsulating them into emulsions to increase their stability and retention of antibacterial activity under the given experimental conditions. Figure 8 shows the quantitative zone-of-inhibition diameters (in mm) for all formulations and the pristine drug solution, used as the reference standard.

**FIGURE 7:**
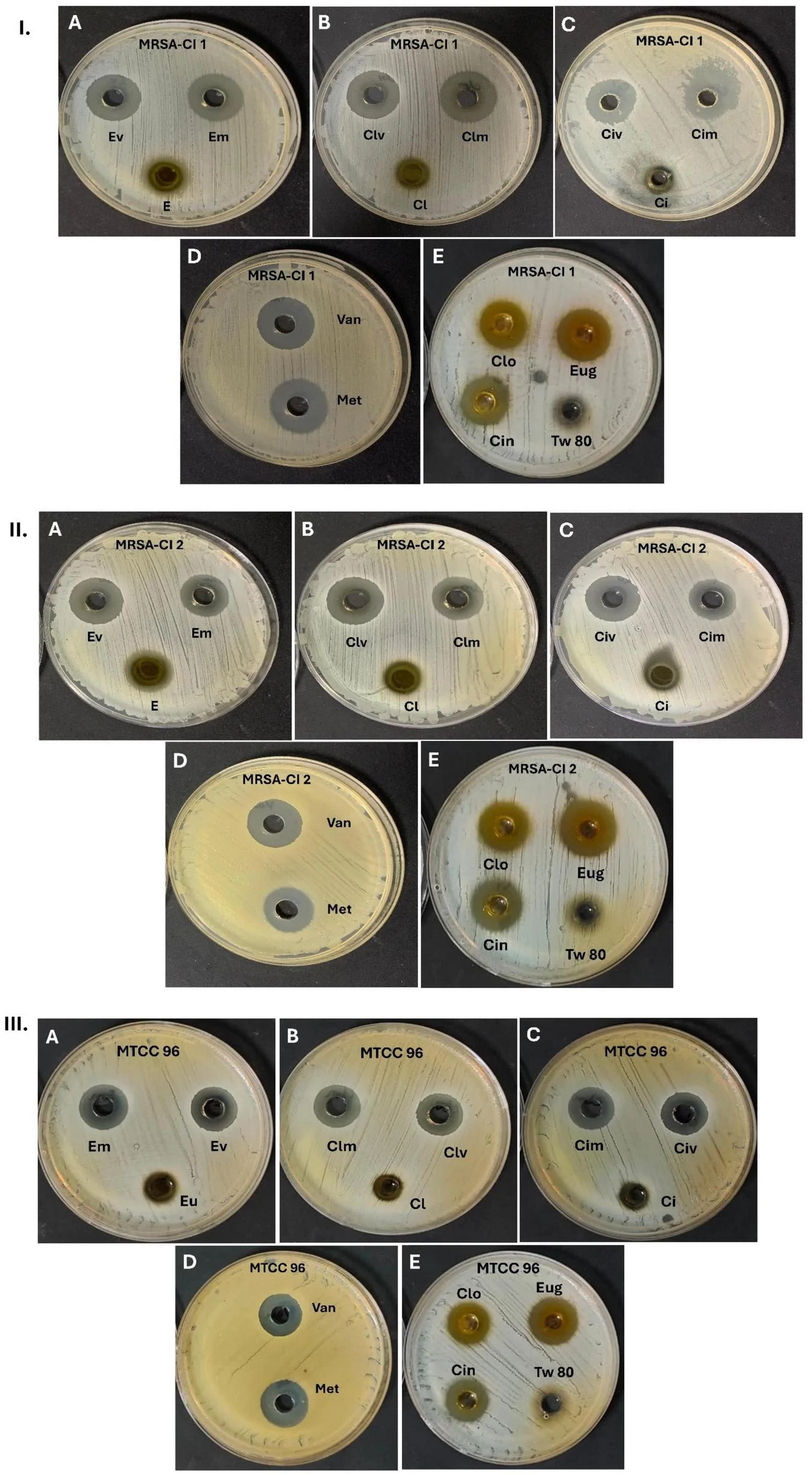
Well diffusion assay results for panel **(A)** Eugenol microemulsions (E – EuME, Ev – Eug-Van, Em – Eug-Met), **(B)** Clove microemulsions (Cl – CLME, Clv – Clo-Van, Clm – Clo-Met), **(C)** Cinnamon microemulsions (Ci – CIME, Civ – Cin-Van, Cim – Cin-Met), **(D)** Pristine drug solutions of Vancomycin and Methicillin, **(E)** Free essential oils and surfactant (Eug – Eugenol, Clo – Clove, Cin – Cinnamon, Tw80 – Tween 80) against **(I)** MRSA-CI 1, **(II)** MRSA-CI 2 and reference strain **(III)** MTCC 96.

**FIGURE 8:**
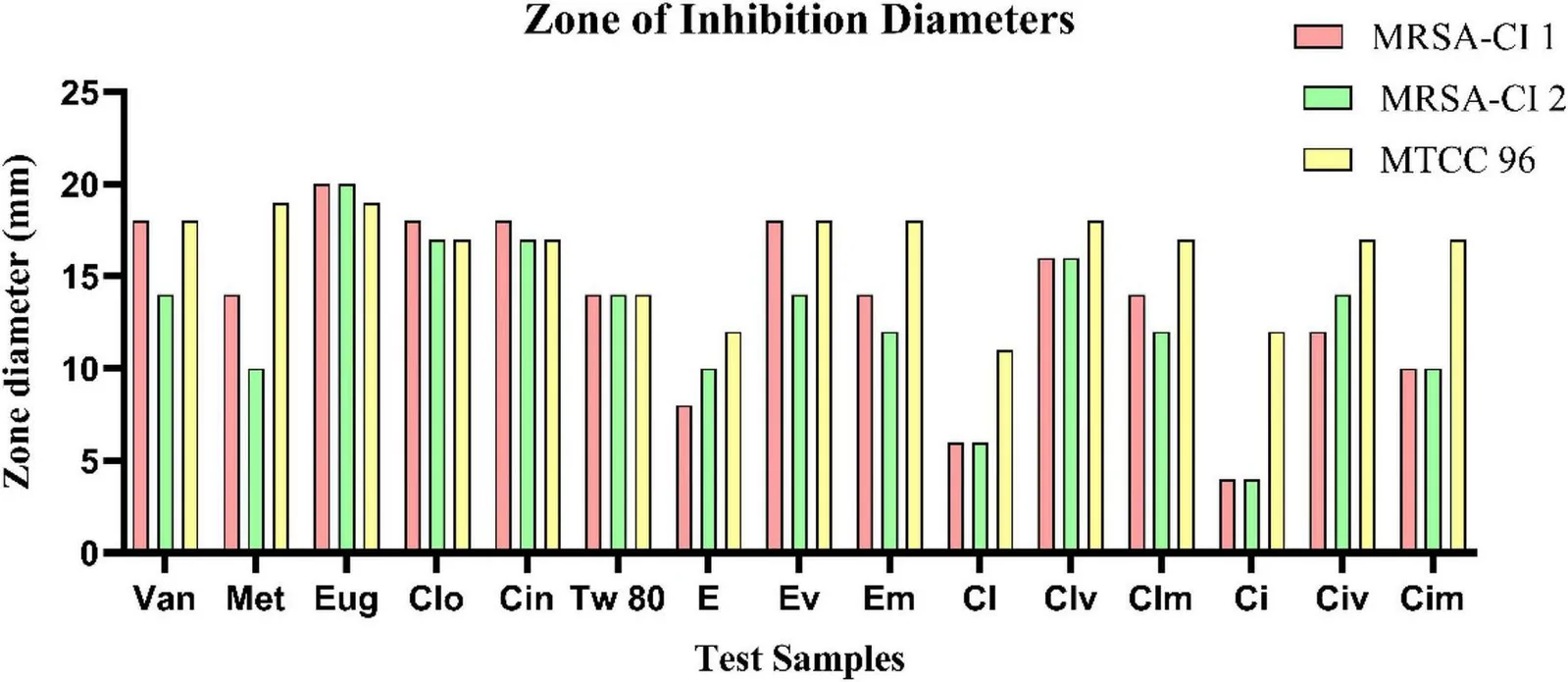
Quantitative values for the zones of inhibition diameters (mm) for the pristine drug solution, free essential oils and surfactant, plain emulsion, and its Vancomycin and Methicillin load modifications against two clinical isolates of MRSA and the reference strain.

#### 3.8.2 Minimum inhibitory concentration

We used MHB medium for all dilutions in the MIC experiment. We determined the antibacterial efficacy of all drug-loaded formulations, including the plain emulsion and pristine drug solutions, using a serial dilution assay against clinical strains MRSA-CI 1, MRSA-CI 2, and the reference strain MTCC 96. For all strains, plain Vancomycin solutions exhibited potent inhibition at 2 μg/mL. In contrast, the Methicillin solution inhibited MRSA-CI 1 at 8 μg/mL, but MRSA-CI 2 and MTCC 96 at 32 μg/mL and 64 μg/mL, respectively. Plain Eugenol emulsion inhibited bacterial growth at 20 μL/mL, 10 μL/mL, and 20 μL/mL. In contrast, for Vancomycin- and Methicillin-loaded moieties, the values were 4 μg/mL, 2 μg/mL, and 2 μg/mL; 8 μg/mL, 8 μg/mL, and 64 μg/mL for MRSA-CI 1, 2, and MTCC 96, respectively. Similarly, the free clove emulsion exhibited MIC values of 20 μL/mL for all the strains. In contrast, in the Vancomycin- and Methicillin-loaded groups, the values were 4 μg/mL, 2 μg/mL, 4 μg/mL, 8 μg/mL, 16 μg/mL, and 64 μg/mL for MRSA-CI 1, MRSA-CI 2, and MTCC 96, respectively. For cinnamon microemulsion, antimicrobial effects occur at concentrations of 20 μL/mL, 10 μL/mL, and 20 μL/mL. At the same time, for the Vancomycin and Methicillin counterparts, the values were 4 μg/mL, 2 μg/mL, 2 μg/mL, and 8 μg/mL, 16 μg/mL, followed by 64 μg/mL for MRSA-CI 1, 2 and MTCC 96, respectively (Table 2). In plain emulsions, Eugenol (EuME) and Cinnamon showed the highest bacterial inhibition at lower concentrations than clove against MRSA-CI 2. In drug-loaded formulations, Clo-Met, followed by Eug-Met, showed potent inhibition at lower concentrations in MRSA-CI 2 and MRSA-CI 1, with a further decrease in concentration required for bacterial inhibition in MRSA-CI 2. Eug-Van, followed by Clo-Van and Cin-Van, showed increased MIC values in MRSA-CI 1. In MRSA-CI 2, concentrations similar to those of the plain Vancomycin solution were required for inhibition. In contrast, the reference strain required concentrations equivalent to the Vancomycin drug solution and much higher than the Methicillin drug solution to produce antibacterial effects.

**TABLE 2:** Antibacterial properties of the optimized drug-loaded emulsions, plain emulsions, pristine drug solutions, free essential oils, and surfactants at the concentration used in the emulsions, with MIC values for drug solutions and drug-incorporated emulsions in μg/mL and oils, surfactants, and plain emulsions in μL/mL.

S. No.	Sample (emulsions)	MIC (μg/mL) and (μL/mL)		
		MRSA-CI 1	MRSA-CI 2	MTCC 96
1.	Vancomycin	2	2	2
2.	Methicillin	8	32	16
3.	Eugenol oil	0.625	0.625	0.625
4.	Clove oil	0.625	0.625	0.625
5.	Cinnamon oil	0.625	0.625	0.625
6.	Tween 80	20	20	20
7.	Eugenol	20	10	20
8.	Vancomycin-eugenol	4	2	2
9.	Methicillin-eugenol	8	8	64
10.	Clove	20	20	20
11.	Vancomycin-clove	4	2	4
12.	Methicillin-clove	8	16	64
13.	Cinnamon	20	10	20
14.	Vancomycin-cinnamon	4	2	2
15.	Methicillin-cinnamon	8	16	64

#### 3.8.3 Screening of biofilm production

The biofilm-forming activity of the clinical isolates and reference strains was evaluated *in vitro*, and results were analyzed based on the classification criterion and OD cutoff value (0.077479). Accordingly, the results showed MRSA-CI 1 as a moderate biofilm former (OD 0.2498 ± 0.06) and clinical strain MRSA-CI 2 and Reference strain MTCC 96 as strong biofilm producers, as indicated by their absorbance values (0.339 ± 0.08, 0.70225 ± 0.17) relative to the OD cutoff. These findings demonstrated the selected strains’ ability to form surface-associated biofilms under the given experimental conditions.

#### 3.8.4 Evaluation of anti-biofilm activity

The anti-biofilm assay determines the lowest concentration of the test compound that substantially reduces established biofilm biomass. The test samples were selected based on good diffusion and MIC results, and the potential of these optimized drug-loaded microemulsions to reduce established biofilms was evaluated. Results demonstrated that concentrations equivalent to MIC values were required to markedly reduce the biofilm biomass (Eug-Van – 4 μg/mL, Eug-Met – 8 μg/mL, Clo-Van - 4 μg/mL, Clo-Met - 8 μg/mL and Cin-Met – 8 μg/mL), except for the Cin-Van formulations in case of MRSA-CI 1, where a reduction in concentration (2 μg/mL) was observed from the MIC value. In the case of MRSA-CI 2, concentrations equivalent to MIC values were sufficient to reduce crystal violet-stained attached biomass for the Eug-Van, Clo-Met, and Cin-Met (2 μg/mL, 16 μg/mL, 16 μg/mL) formulations; however, for the Eug-Met, Cin-Van, and Clo-Van formulations, an increase in concentration (16 μg/mL, 4 μg/mL, 8 μg/mL) was required to substantially reduce the biofilm biomass. For the reference strain, there was a marked reduction in the concentrations of all the drug-loaded microemulsions (Eug-Met – 8 μg/mL, Clo-Van – 1 μg/mL, Clo-Met, and Cin-Met – 8 μg/mL) required to minimize the biofilm biomass in comparison to the MIC values (Table 3).

**TABLE 3:** Antibacterial properties of the optimized drug-loaded emulsions and their biofilm reduction endpoint values in comparison to the respective MIC values in μg/mL.

S. No.	Sample (emulsions)	MIC (μg/mL)			Biofilm reduction endpoint (μg/mL)		
		MRSA-CI 1	MRSA-CI 2	MTCC 96	MRSA-CI 1	MRSA-CI 2	MTCC 96
1.	Eug-Van	4	2	2	4	2	8
2.	Eug-Met	8	8	64	8	16	8
3.	Clo-Van	4	2	4	4	8	1
4.	Clo-Met	8	16	64	8	16	8
5.	Cin-Van	4	2	2	2	4	8
6.	Cin-Met	8	16	64	8	16	8

#### 3.8.5 Evaluation of checkerboard assay

The checkerboard assay was used to determine the effects of combining plain emulsions and pristine drug solutions against clinical isolates of MRSA and the MTCC strain. We evaluated interactions between the test components using the Fractional Inhibitory Concentration Index (FICI). The components analyzed were plain Eugenol emulsion, clove emulsion, and cinnamon emulsion with vancomycin and methicillin drug solutions. The results showed FICI values of 0.375, 0.25, 0.625, 0.375, 0.625, and 0.625 for the EuME+Van, EuME+Met, CLME+Van, CLME+Met, CIME+Van, and CIME+Met formulations, respectively, against MRSA-CI 1, thereby classifying them as Synergistic (FICI ≤ 0.5) and additive (0.5 < FICI ≤ 1). For MRSA-CI 2, similar interactions were observed, with FICI values of 0.375, 0.25, 0.625, 0.375, 0.625, and 0.375 for the EuME+Van, EuME+Met, CLME+Van, CLME+Met, CIME+Van, and CIME+Met formulations, respectively. However in case of MTCC 96, the interactions were found to be Indifferent (1 < FICI ≤ 4), in case of vancomycin formulations (EuME+Van, CLME+Van, CIME+Van) with FICI values being 1.125 and Synergistic for Methicillin loaded formulations with FICI values being 0.25 for EuME+Met and 0.375 for CLME+Met, and CIME+Met formulations, respectively (Table 4).

**TABLE 4:** Fractional Inhibitory Concentration Indices (FICI) and their interpretation for the combinations of pristine drug solutions (A) and plain microemulsions (B), their MIC alone (drug solutions - μg/mL, plain emulsions - μL/mL), MIC in combination, and their respective Fractional Inhibitory Concentration.

Strains	Formulations	MIC alone	MIC in combination	FIC A	FIC B	FICI	Interpretation
MRSA-CI 1	Van+EuME	2 μg/mL, 20 μL/mL	0.5 μg/mL, 2.5 μL/mL	0.25	0.125	0.375	Synergy
	Met+EuME	8 μg/mL, 20 μL/mL	1 μg/mL, 2.5 μL/mL	0.125	0.125	0.25	Synergy
	Van+CLME	2 μg/mL, 20 μL/mL	1 μg/mL, 2.5 μL/mL	0.5	0.125	0.625	Additive
	Met+CLME	8 μg/mL, 20 μL/mL	2 μg/mL, 2.5 μL/mL	0.25	0.125	0.375	Synergy
	Van+CIME	2 μg/mL, 20 μL/mL	1 μg/mL, 2.5 μL/mL	0.5	0.125	0.625	Additive
	Met+CIME	8 μg/mL, 20 μL/mL	4 μg/mL, 2.5 μL/mL	0.5	0.125	0.625	Additive
MRSA-CI 2	Van+EuME	2 μg/mL, 10 μL/mL	0.5 μg/mL, 1.25 μL/mL	0.25	0.125	0.375	Synergy
	Met+EuME	32 μg/mL, 10 μL/mL	4 μg/mL, 1.25 μL/mL	0.125	0.125	0.25	Synergy
	Van+CLME	2 μg/mL, 20 μL/mL	1 μg/mL, 2.5 μL/mL	0.5	0.125	0.625	Additive
	Met+CLME	32 μg/mL, 20 μL/mL	8 μg/mL, 2.5 μL/mL	0.25	0.125	0.375	Synergy
	Van+CIME	2 μg/mL, 10 μL/mL	1 μg/mL, 1.25 μL/mL	0.5	0.125	0.625	Additive
	Met+CIME	32 μg/mL, 10 μL/mL	8 μg/mL, 1.25 μL/mL	0.25	0.125	0.375	Synergy
MTCC 96	Van+EuME	2 μg/mL, 20 μL/mL	2 μg/mL, 2.5 μL/mL	1	0.125	1.125	Indifference
	Met+EuME	16 μg/mL, 20 μL/mL	2 μg/mL, 2.5 μL/mL	0.125	0.125	0.25	Synergy
	Van+CLME	2 μg/mL, 20 μL/mL	2 μg/mL, 2.5 μL/mL	1	0.125	1.125	Indifference
	Met+CLME	16 μg/mL, 20 μL/mL	4 μg/mL, 2.5 μL/mL	0.25	0.125	0.375	Synergy
	Van+CIME	2 μg/mL, 20 μL/mL	2 μg/mL, 2.5 μL/mL	1	0.125	1.125	Indifference
	Met+CIME	16 μg/mL, 20 μL/mL	4 μg/mL, 2.5 μL/mL	0.25	0.125	0.375	Synergy

#### 3.8.6 Evaluation of killing kinetics

Figure 9 shows the killing kinetics of the three bacterial isolates upon treatment with the drug-loaded microemulsions at 2× MIC concentrations. In MRSA-CI 1, for the vancomycin-loaded formulations, Eug-Van showed an early reduction in viable bacterial count compared with the growth control (GC) from the second hour, with a 2.04 log10 decrease that increased to 2.27 log10 by the 8th hour, indicating progressive antimicrobial activity. For Clo-Van, similar results were observed, and time-dependent killing was prominent, as the reduction in viable counts increased from 1.55 log10 at the 2nd hour to 2.63 log10 by the 8th hour. For Cin-Van emulsions, a gradual rise in antibacterial activity was observed, wherein a marked increase in reduction of viable counts was observed from the 2nd hour with 1.60 log10 viable colonies to 2.33 log10 reductions at the 8th hour, indicating prolonged exposure could enhance antibacterial activity, wherein all the 3 formulations showed no colonies at the 24th hour, showing marked reduction in viable counts under tested conditions. For Methicillin formulations, Eug-Met showed a marked rise in antibacterial activity by the 6th hour, with 2.75 log10 reductions from the growth control and a substantial reduction of viable colonies by the 8th hour. For Clo-Met, a conspicuous difference was observed: the reduction in viable colonies reached 3.33 log10 compared to the growth control by the 8th hour; however, for the Cin-Met formulations, a delayed antibacterial effect was observed, with minimal difference until the 6th hour, and a prominent reduction in viable colonies was detected by the 24th hour under the given experimental conditions. For MRSA-CI 2, Eug-Van showed time-dependent progression in antibacterial activity, with a marked decrease in viable counts of 1.89 log10 by the 8th hour; for Clo-Van and Cin-Van, a prominent reduction was observed only by the 6th hour, indicating slow but progressive enhancement of antimicrobial activity. For Eug-Met, Clo-Met, and Cin-Met, a prominent reduction in viable count was observed only by the 6th hour, which increased further, demonstrating sustained inhibition dependent on longer exposure time. For MTCC 96, the Vancomycin formulations showed antibacterial effects earlier, with progressive and sustained effects throughout the study duration; among the Methicillin formulations, Eug-Met had a more pronounced effect in reducing viable bacterial counts, while Clo-Met and Cin-Met showed a more sustained effect than the growth control.

**FIGURE 9:**
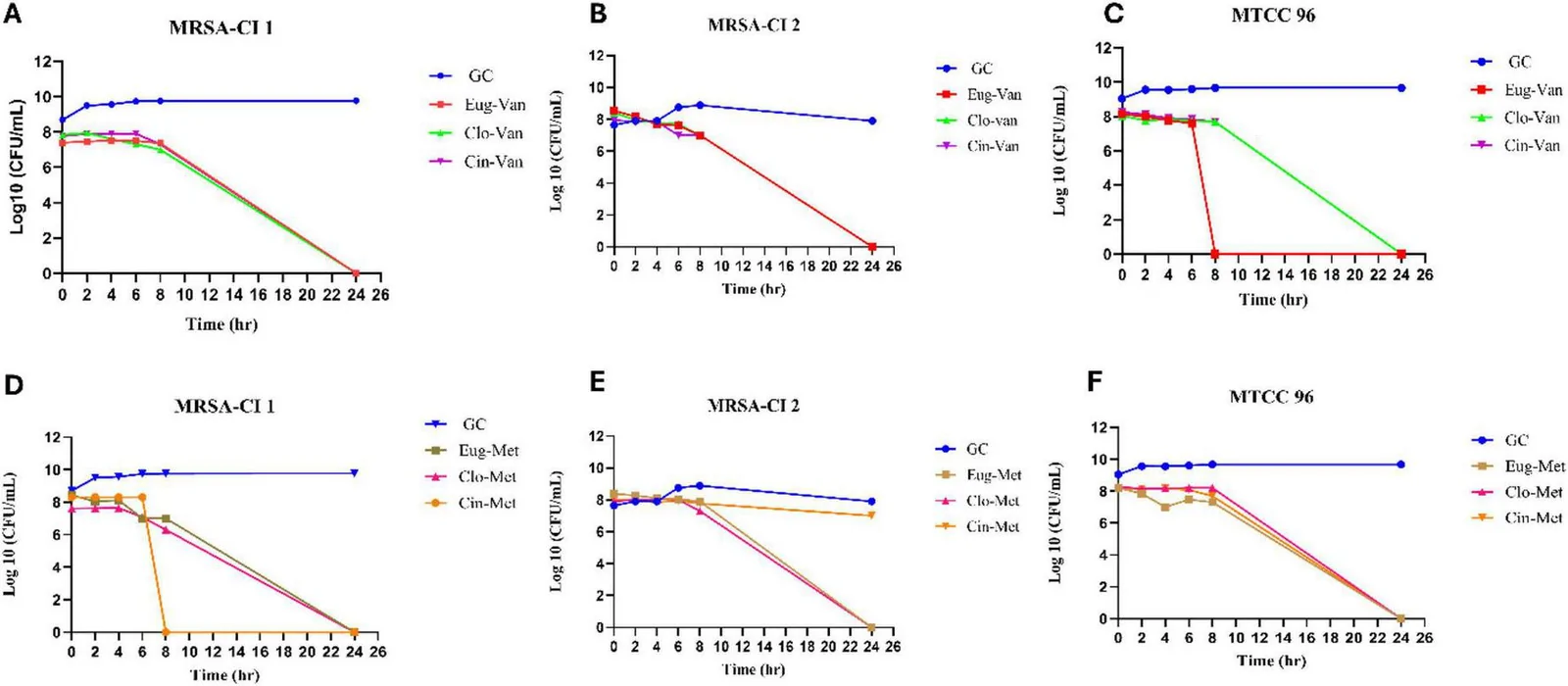
Kinetics of killing by the vancomycin-loaded emulsions (Eug-Van, Clo-Van, Cin-Van) and methicillin-loaded emulsions (Eug-Met, Clo-Met, Cin-Met) at their 2× MIC concentration for the clinical isolates MRSA-CI 1 **(A,D)**, MRSA-CI 2 **(B,E)**, and reference strain MTCC 96 **(C,F)**, respectively.

### 3.9 Assessment of entrapment efficiency

Evaluation of the entrapment efficiency of the drug-loaded microemulsions revealed the respective encapsulation values of the drugs within the microemulsion system. The results showed encapsulation values of 71.4%, 74.9%, and 78.2% for Vancomycin in the Eug-Van, Clo-Van, and Cin-Van formulations. In contrast, values were 52.2%, 54.1%, and 56.9% for Methicillin in the Eug-Met, Clo-Met, and Cin-Met formulations, respectively.

### 3.10 Release kinetics assay

Figures 10A,B show the diffusion patterns of Vancomycin and Methicillin across all the drug-loaded emulsions. The release kinetics show a slow, controlled release over 24 h, demonstrating sustained drug diffusion from the carrier emulsions. For the Vancomycin-loaded emulsions, Eug-Van, Clo-Van, and Cin-Van had cumulative drug release of 98.81% ± 1.14%, 52.32% ± 0.12%, and 97.91% ± 0.08%, respectively, over 24 h. In contrast, for the Methicillin-encapsulated emulsions, Eug-Met, Clo-Met, and Cin-Met, the emulsions released the drug at cumulative concentrations of 72.01% ± 0.56%, 58.24% ± 2.19%, and 27.47% ± 0.14%, respectively. This pattern of gradually increasing release until the appearance of a saturation region indicates controlled release pattern which can help to retain effective local concentrations of the therapeutics at the site of action for extended duration thereby lowering burst release and enhancing patient compliance while inhibiting bacterial growth over longer durations.

**FIGURE 10:**
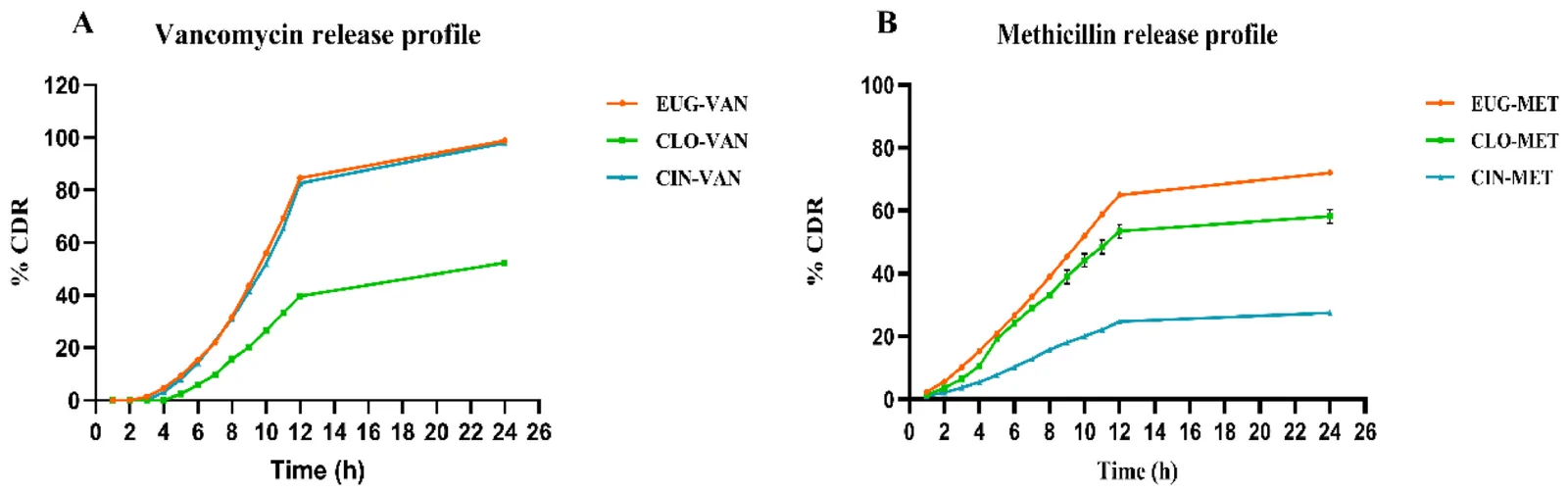
Drug release profiles for Vancomycin hydrochloride from panel **(A)** Vancomycin-loaded microemulsions – EUG-VAN, CLO-VAN, and CIN-VAN microemulsions and Methicillin Sodium salt from panel **(B)** Methicillin-loaded microemulsions - EUG-MET, CLO-MET, and CIN-MET over the period of 24 h, values represented as Mean ± SD, *n* = 3, error bars are smaller than data symbols.

## 4 Discussion

Microemulsions are optically clear, translucent, thermodynamically stable colloidal dispersion systems comprising both water-soluble and lipid-soluble phases (Dhaval et al., 2022). Their popularity as advanced localized drug-delivery platforms for delivering therapeutic drugs and bioactive molecules via various routes of administration has been gaining attention. Such emulsion systems exhibit smaller droplet sizes and narrower PDI distributions, indicating greater homogeneity, longer storage lifespans, enhanced thermal and kinetic stability, greater interfacial area (Jacob et al., 2024), and improved solubility and drug diffusivity (Izadiyan et al., 2025), even with minimal energy requirements. The choice of therapeutic agents included Vancomycin and Methicillin, with Vancomycin recognized as a powerful antimicrobial glycopeptide primarily used to treat Gram-positive infections and considered the gold-standard, first-line drug for treating MRSA infections (Cong et al., 2020). Methicillin, on the other hand, was the first semisynthetic penicillinase-resistant antimicrobial (Castle, 2007) and is effective against many Gram-positive strains, including *Staphylococcus aureus*.

Both the plain and drug-loaded microemulsions showed no creaming, flocculation, or turbidity. All the different formulations displayed the optimal nanoscale size range (15–30 nm), while the PDI being <0.5 indicated high homogeneity and uniform dispersion of the particles within the medium (Tungadi, 2024). These results established the formulations’ colloidal stability, homogeneous distribution, and enhanced penetration into complex microbial environments due to their nanoscale size range compared to high interfacial area, thereby facilitating their improved permeation across biological membranes. This effective droplet diameter range aligns with a previous study by Haq et al. (2023), which prepared a curcumin-based Nanoemulsion to test its antimicrobial properties. In contrast, the monodispersity of these emulsions is consistent with a recent study by Gholizadeh et al. (2026), which examined the antibiofilm effects of Cinnamon Nanoemulsion. At the same time, Zeta potential values indicate that the synthetic non-ionic surfactant Tween 80 forms a protective layer around the particles, promoting interaction between the oil and water phases and hindering clustering through steric effects, thereby promoting colloidal stability (Debraj et al., 2023). Tabaniag et al. (2023) reported similar observations, using soy lecithin as a surfactant in an emulsion system to prevent droplet agglomeration. The zeta potential values were negative but within the acceptable limits of ±30 mV, with more negative values indicating a more stable emulsion system due to strong repulsive forces between droplets and possible steric effects from the non-ionic surfactant Tween 80, thereby preventing aggregation. An earlier study (Jahromy et al., 2024) reported a curcumin nanoemulsion for the treatment of dry eyes with a similar zeta potential. All 9 formulations remained thermodynamically stable after freeze-thaw and heat-cool cycles. This stress testing is essential for determining the storage and shelf life of the emulsions over longer periods, with minimal changes in separation. This aligns with (Ali et al., 2014), in which thermodynamic stability testing determined optimal stability and the potential for further characterization of the dutasteride-loaded Nanoemulsion. The pH range for all emulsions was 5.0–6.0, mostly in the acidic region. This could be attributed to the presence of numerous acidic moieties, such as -COOH and -C = O, which also explain their negative surface charge densities. The drug-loaded emulsions had slightly higher pH values, and the reduced negative charge may be attributed to interactions between the drugs and the lipid or aqueous phases of the emulsions (Mahor et al., 2023). Healthy human skin has a pH of around 5; thus, the acidic pH of the emulsions is essential for maintaining the acidic cloak of healthy skin while preventing microbial growth in the wound bed. A study by Hayat et al. (2025) found that the acidic pH of the Nanoemulsion and its cream was essential for maintaining hydration and antimicrobial properties. The FTIR spectra for the pristine emulsion and its two drug-modified variations were checked for the presence of respective functional groups and bond vibrations. All the spectra displayed prominent peaks for the respective essential oils; Eugenol which was in accordance with the study by Yang et al. (2019) for separation of Eugenol using beta cyclodextrin, for Clove similar peaks were observed in a study by Jose et al. (2022) where clove oil and chitosan nanoparticle was used in the formulation of a PVA based nanocomposite, and for Cinnamon, a study by Tambe and Gotmare (2022) revealed similar range of peaks in case of cinnamon oil extracted by hydro distillation. Presence of distinct peaks for the surfactant Tween 80, correlated to a study by Bisht et al. (2022) wherein Tween 80 was used to maintain stability of carbon nanotubes and graphene. Moreover, the spectra exhibited broadened characteristic peaks for the drug-loaded modifications, compared with the plain emulsion spectra, especially for Vancomycin and Methicillin, which were in accordance with the studies by Martín et al. (2021) and Wilkinson et al. (1999), which observed the characterization of the antibiotic hosted by bentonite and the enzymatic inactivation of Methicillin Sodium salt by the beta-lactam-degrading enzymes sourced from Citrobacter. These results confirmed the successful incorporation of all elements into the emulsion blend, with no disappearance of distinct characteristic peaks or corresponding peak shifts due to intermolecular exchange, thus indicating their chemical stability. The TEM micrographs showed spherical to oval droplets that appeared dark. However, the average diameters differed considerably from the DLS diameters because TEM measurements capture the physical dimensions of individual droplets, which are subject to sample preparation and drying. In contrast, DLS measures the particle’s average hydrodynamic diameter. These differences may stem from differences in measurement parameters and principles, such as DLS calculating diameters for diluted, dispersed droplets versus TEM analyzing specific core droplets. The appearance and morphology of the droplets within the emulsions, and the difference between the hydrodynamic and TEM diameters, could be correlated with the study by Hashem et al. (2024), which reported similar results in clove and thyme nanoemulsion formulations.

Antimicrobial characterization of the limited number of clinical isolates was based on MRSA status designated by the source laboratory; therefore, the present study did not include independent molecular validation or explicit confirmation of resistance profiles (*mecA* or PBP2a status). However, among the antimicrobial assays conducted in this investigation, larger zones of inhibition in the well-diffusion assay indicate greater antimicrobial potential. In this case, the greatest zones of inhibition were recorded for the Eug-Van, followed by the Clo-Van and Cin-Van formulations, compared to the Methicillin counterparts – in the order of Clo-Met, Eug-Met, and Cin-Met emulsions under the given plating conditions. Thus, in both cases, Vancomycin-loaded modifications showed the most potent inhibition with Eug-Van and Clo-Van in MRSA-CI 1 and 2. At the same time, the emulsion-incorporated methicillin formulations, Clo-Met and Eug-Met, showed the strongest effect against both the tested clinical strains. In MTCC 96, drug-loaded formulations with Eugenol produced larger zones of inhibition, followed by Clove and Cinnamon emulsions encapsulating the drug solutions. However, for free essential oils, much larger zones of inhibition were observed against all 3 strains than for their plain emulsions, which could be attributed to reduced viscosity and unrestricted diffusion due to the absence of encapsulation. The zones of inhibition, though small, were observed for the plain emulsions, indicating their inherent antibacterial activity, and maintained the same order: Eugenol exhibited the greatest activity, followed by clove and Cinnamon. In another study by Hayat et al. (2025), they observed enhanced antibacterial activity when the phytoextract was incorporated into the emulsion formulation. Manzoor et al. (2023) demonstrated greater inhibition with microemulsions of essential oils and attributed this activity to enhanced diffusion of the active constituents to the site of action via penetration through the aqueous-loving phase of the nanovesicles. The well diffusion assay for pristine drug solutions was used as the standard, with zones of 18 mm, 14 mm, and 18 mm for Vancomycin and 14 mm, 10 mm, and 19 mm for Methicillin in MRSA-CI 1, MRSA-CI 2, and MTCC 96, respectively, indicating greater effectivity with the drug-incorporated emulsion formulations.

In the broth microdilution method, the plain Vancomycin solution was effective at 2 μg/mL against all strains; however, a higher concentration was required for bacterial inhibition with the Methicillin solution, reaching 32 μg/mL against MRSA-CI 2. For plain emulsions, Eugenol and cinnamon showed the most effective concentration at 10 μL/mL against MRSA-CI 2; for clove, inhibition was more pronounced at 20 μL/mL against all strains. In vancomycin-loaded Eugenol formulations, only MRSA-CI 1 was inhibited at a higher concentration of 4 μg/mL. In contrast, inhibition occurred at a comparatively lower concentration of 2 μg/mL in MRSA-CI 2 and MTCC 96. For the vancomycin-loaded clove emulsion, effects similar to those observed with Eug-Van were observed for MRSA-CI 1 and MTCC 96, with inhibition occurring at a higher concentration of 4 μg/mL for each. For cinnamon-vancomycin counterparts, drug-loaded forms showed similar effects, in accordance with the results for Eugenol-loaded vancomycin emulsions for MRSA-CI 1. In contrast, for MRSA-CI 2, inhibition occurred at a lower concentration (2 μg/mL). For the Methicillin-loaded moieties MRSA-CI 1, Eug-Met, Clo-Met, and Cin-Met, all showed inhibition at 8 μg/mL. Against MRSA-CI 2, Cin-Met and Clo-Met inhibited bacterial growth at 16 μg/mL. In contrast, Eug-Met showed inhibition at a comparatively lower concentration, with effects similar to MRSA-CI 1. In contrast, in MTCC 96, all formulations showed inhibition at a higher concentration of 64 μg/mL. However, the free oils had considerably lower MIC values than their emulsion formulations against all strains. These results were consistent with the study by Seifi et al. (2026), wherein although Cinnamon essential oil had lower MIC values, its nano formulation showed improved activity and better stability in interaction with Fluconazole. Although the tested formulations showed different MIC values, direct potency comparisons between the antibiotics and free essential oils could not be made because their concentrations were expressed in non-equivalent units. However, the activity of complete emulsion should be considered as a combined outcome of the constituent components and their respective physicochemical attributes. The free oils and surfactants demonstrated intrinsic antibacterial activity upon independent evaluation but at their respective undiluted test concentrations and thus do not establish the inferiority of the complete formulation. Moreover, the emulsion formulations retained comparable antibacterial effects even when these components were incorporated at substantially lower concentrations than the corresponding free controls, thereby indicating the advantage of emulsion-mediated dispersion rather than an intrinsic increase in antibacterial efficacy. Accordingly, smaller dispersion domains and a larger interfacial area might favor distribution within complex microbial environments. At the same time, achieving antimicrobial activity at lower quantities of test components may reduce potential toxicity and host-cell incompatibility caused by exposure to high concentrations, demonstrating formulation advantages. However, this requires further experimental validation to determine concentration-dependent antimicrobial and cytocompatibility profiles of the complete formulations.

Biofilm screening demonstrated the selected bacterial isolates’ potential to form established biofilms under the experimental conditions. Classifying biofilm producers as strong, moderate, or weak is crucial in wound-associated infections such as DFUs, as established biofilms can substantially reduce antibacterial susceptibility while progressively increasing tolerance to antimicrobial therapeutics compared to free planktonic cells (Dalar and Gönüllü, 2026). These biofilm matrices hinder antimicrobial penetration, while mechanisms like efflux pumps further reduce susceptibility by effectively pumping out compounds (Sorenson et al., 2025). Thus, strains that form stronger biofilms may require higher concentrations or sustained exposure to antimicrobials to disrupt their activity. The findings showed that the selected strains can form surface-associated biofilms. They were categorized as moderate and strong biofilm producers based on predefined criteria under the experimental conditions, thereby supporting their use to evaluate the antibiofilm activity of the drug-loaded microemulsions. We evaluated the potential of the optimized formulations to reduce established biofilms using the biofilm reduction endpoint assay, which is highly relevant to pathogen-associated wound infections, where resident planktonic microbes develop persistent biofilm matrices that resist antimicrobial therapeutics. We analyzed the results to determine the minimum concentration of the test formulations capable of reducing biofilm mass, characterized by enhanced antimicrobial tolerance, and interpreted them alongside MIC values to compare endpoints; values lower than or equivalent to the MIC concentration could indicate better activity of the test samples. Results showed that concentrations equivalent to MIC values were required to reduce biofilm biomass, except for the Cin-Van formulations in MRSA-CI 1, where a lower concentration reduced biofilm biomass. In MRSA-CI 2, concentrations equivalent to the MIC were sufficient to reduce biofilms for the Eug-Van, Clo-Met, and Cin-Met formulations; however, for the Eug-Met, Clo-Van, and Cin-Van formulations, higher concentrations were required to reduce crystal violet-stained attached biofilm biomass. A similar result was reported by Shaghayegh et al. (2026), in which *Staphylococcus aureus* biofilms from chronic rhinosinusitis showed considerably higher biofilm-reduction endpoint values than the MIC values against all five tested components, at much lower concentrations to reduce the biofilm biomass. To assess the efficacy of interactions between the selected antibacterial drug components and phytochemical constituents, we performed checkerboard assays. We interpreted the results using Fractional Inhibitory Concentration Indices (FICI) to quantitatively assess the combined effect of the constituents compared with their individual antimicrobial effects; lower concentrations of the individual components required to inhibit bacterial growth upon combination indicate enhanced antimicrobial activity. The results for the clinical isolates demonstrated synergistic activity, indicating enhanced antibacterial effect upon combination, with additive effects dictating their subsequent individual complementary contribution to antimicrobial effects without crossing the threshold of synergy. A study by Hassan et al. (2025) demonstrated similar synergistic effects with carvacrol gelatin nanoemulsions and terbinafine; in contrast, the interaction was additive for Eugenol and methyl Eugenol gelatin Nanoemulsions in combination with Terbinafine. However, for the reference strain, Vancomycin in combination with the phytochemical components showed an indifferent effect demonstrating that there was no substantial augmentation in the antibacterial effects upon the combination of these components wherein similar effects were observed in a study by Ribeiro et al. (2024) where macela extract incorporated into flaxseed Nanoemulsion was tested in combination with antibiotics such as oxacillin and Penicillin. However confirmation of the antibacterial activity should be validated through other mechanistic assay like time kill assays. The time-kill assays showed a time-dependent reduction in viability of the selected bacterial isolates when tested against optimized drug-loaded formulations at 2× MIC concentrations. The results indicated a marked reduction in viable bacterial colonies in the treated groups compared to the growth control, with the difference progressively increasing with sustained exposure to the antimicrobial formulations. In contrast, the untreated control maintained stable viable colonies throughout the experiment, indicating that colony reduction was associated with the treatment modalities. However, these results may reflect *in vitro* activity, and their relevance to potential application in wound management requires further mechanistic investigation. Almost all test formulations substantially reduced viable counts under the tested conditions by the 24th hour, complementing the MIC findings. However, Cin-Met, although reduced viable colonies compared with the growth control, did not produce a substantial reduction even after 24 h against the clinical isolate MRSA-CI 2. These differences in activity may be attributed to the specific interaction between the drug and phytoconstituent combination and its activity against the tested bacterial isolate. In contrast, a study reported by El-Sherbiny et al. (2024) showed effective reduction of viable colonies using cinnamon oil Nanoemulsion against colistin-resistant *Klebsiella pneumoniae* at half the MIC concentration. Evaluating the entrapment efficiency of drug-loaded microemulsions assesses the extent of drug entrapment and retention within the emulsion matrix. For both drug counterparts, the cinnamon emulsion showed the greatest entrapment efficacy, where a higher value indicates successful incorporation into the emulsions and their adequacy as reservoirs for sustained, prolonged release. However, compared with Methicillin formulations, Vancomycin formulations had greater potential for drug entrapment where this variation could be attributed to differences in the physicochemical properties of the encapsulated drugs, their aqueous solubility and stability, and their respective affinities for the lipid-surfactant interface. Lower encapsulation efficiency could be attributed to hydrophilic drugs that remain in the continuous water phase or at the oil-water interface rather than in the lipid matrix which was consistent with findings by Esen Yigit and Lamprecht (2026). Although these findings indicate the feasibility of encapsulating these antimicrobial agents within oil-based emulsion systems, the contribution of individual formulation components and the formulation structure should be considered when interpreting the observed effects. For the drug release profiles, all formulations showed controlled, sustained release, with increasing concentrations over 24 h. In the case of Vancomycin-loaded moieties, Eug-Van had the highest cumulative drug release of around 98% over 24 h. In contrast, for the Methicillin-loaded ones, similar results were observed: Eug-Met (72%) had the highest release percentage. The drug-loaded emulsions exhibited a gradual increase in drug concentration over 12 h. They plateaued around 24 h, demonstrating sustained release over a long period. Thus, incorporating these drugs within the microemulsion carriers could enhance their potential for antimicrobial activity at the definite site of action to augment their efficacy over prolonged durations by reducing dosage frequency, with potential for enhanced localized delivery by the absence of sudden release immediately after application. This in turn might facilitate the capability to retain the antibacterial effects throughout the complete duration of healing in relevant wound models through prolonged availability of these antimicrobial formulations at the specific site of application. However, the present investigation is limited to the physicochemical and *in vitro* antimicrobial evaluation of the optimized formulations. Although the strategy for their development was motivated by its potential relevance in future investigations of MRSA infection-associated diabetic wounds, the potential efficacy of wound management, probable cytocompatibility and systemic safety, and therapeutic effectiveness were not directly evaluated. Thus further *in vitro* and *in vivo* studies are required to validate and establish their potential biological safety and preclinical efficiency.

## 5 Conclusion

In this research effort, Vancomycin- and Methicillin-encapsulated Eugenol, Clove, and Cinnamon microemulsions were formulated and optimized to investigate their physicochemical stability, retention of antibacterial properties, and controlled release of therapeutics to the site of action against two clinical MRSA isolates, MRSA-CI 1 and MRSA-CI 2, and a reference strain, MTCC 96. The optically clear oil-in-water formulation was further physicochemically characterized. Based on the experimental outcomes, it can be inferred that the optimized drug-loaded microemulsions were stable and, upon antibacterial testing, demonstrated equivalent and potent antimicrobial activity with the drug-loaded emulsions compared to the free drug solution or plain emulsion, specifically the Vancomycin formulations (Eug-Van and Clo-Van) against MRSA-CI 1 and MRSA-CI 2, respectively. In contrast, Methicillin formulations, especially Clo-Met and Cin-Met, demonstrated measurable *in vitro* antimicrobial activity against MRSA-CI 2 strains, respectively, under test conditions. The drug-encapsulated formulations showed controlled drug release over 24 h, with maximum drug release in the case of Eug-Van formulations. These findings demonstrate substantial potential for these drug-loaded microemulsions to produce measurable *in vitro* antimicrobial activity against the tested clinical isolates, wherein the observed formulation-dependent and isolate-dependent results should be interpreted in the context of the intrinsic contribution of individual components. Thus, these physicochemical and antimicrobial findings support investigation of their potential for localized antimicrobial delivery to potentially combat MRSA infections in chronic diabetic wounds. However, further preclinical and clinical investigations are needed to support future perspectives of wound-management applications and to determine human-specific dose-dependent pharmacodynamics and pharmacokinetics.

## Data Availability

The original contributions presented in this study are included in this article/supplementary material, further inquiries can be directed to the corresponding author.

## References

[B1] Adekanmbi A. O. Adesola R. O. Adeyemi A. O. Mbionwu C. C. (2023). “The threat of methicillin resistant *Staphylococcus aureus* (MRSA) in the aquatic environment via wastewater generated from healthcare facilities,” in *Bacterial Infectious Diseases Annual Volume*, eds Garbacz K. Jarzembowski T. A. Rodriguez-Morales A. J. (London: IntechOpen). 10.5772/intechopen.113967

[B2] Ali M. S. Alam M. S. Alam N. Siddiqui M. R. (2014). Preparation, characterization and stability study of dutasteride loaded nanoemulsion for treatment of benign prostatic hypertrophy. *Iran. J. Pharm. Res.* 13, 1125–1140. 10.22037/ijpr.2014.1563 PMC4232777 25587300

[B3] Ambade S. S. Gupta V. K. Bhole R. P. Khedekar P. B. Chikhale R. V. (2023). A review on five and six-membered heterocyclic compounds targeting the Penicillin-binding protein 2 (PBP2A) of methicillin-resistant *Staphylococcus aureus* (MRSA). *Molecules* 28:7008. 10.3390/molecules28207008 37894491 PMC10609489

[B4] Asiri Y. I. Venkatesan K. (2025). Wound-healing potential of *Myristica fragrans* essential oil: A multi-targeted approach involving inflammation, oxidative stress, and apoptosis regulation. *Pharmaceuticals* 18:880. 10.3390/ph18060880 40573275 PMC12195913

[B5] Baltzis D. Eleftheriadou I. Veves A. (2014). Pathogenesis and treatment of impaired wound healing in diabetes mellitus: New insights. *Adv. Ther.* 31, 817–836. 10.1007/s12325-014-0140-x 25069580

[B6] Bisht A. Zuñiga-Bustos M. Prasher G. Gautam S. Poblete H. Singh R. P. (2022). Stabilization of carbon nanotubes and graphene by tween-80: Mechanistic insights from spectroscopic and simulation studies. *Langmuir* 38, 10173–10182. 10.1021/acs.langmuir.2c01190 35947770

[B7] Castle S. S. (2007). “Methicillin,” in *xPharm: The Comprehensive Pharmacology Reference*, eds Enna S. J. Bylund D. B. (Amsterdam: Elsevier), 1–4. 10.1016/B978-008055232-3.62147-6

[B8] Cong Y. Yang S. Rao X. (2020). Vancomycin resistant *Staphylococcus aureus* infections: A review of case updating and clinical features. *J. Adv. Res.* 21, 169–176. 10.1016/j.jare.2019.10.005 32071785 PMC7015472

[B9] Dalar Z. G. Gönüllü N. (2026). Biofilm-associated reduced antimicrobial susceptibility in *Pseudomonas aeruginosa* isolates from cystic fibrosis patients: MIC–MBEC discordance. *Diagn. Microbiol. Infect. Dis.* 116:117577. 10.1016/j.diagmicrobio.2026.117577 42531940

[B10] Debraj D. Carpenter J. Vatti A. K. (2023). Understanding the Effect of the oil-to-surfactant ratio on eugenol oil-in-water nanoemulsions using experimental and molecular dynamics investigations. *Ind. Eng. Chem. Res.* 62, 16766–16776. 10.1021/acs.iecr.3c02345

[B11] Deng H. Li B. Shen Q. Zhang C. Kuang L. Chen R. et al. (2023). Mechanisms of diabetic foot ulceration: A review. *J. Diabetes* 15, 299–312. 10.1111/1753-0407.13372 36891783 PMC10101842

[B12] Dhaval M. Vaghela P. Patel K. Sojitra K. Patel M. Patel S. et al. (2022). Lipid-based emulsion drug delivery systems — A comprehensive review. *Drug Deliv. Transl. Res.* 12, 1616–1639. 10.1007/s13346-021-01071-9 34609731

[B13] Dighriri I. M. Alanazi S. AlMutairi K. Alhusayni S. J. Balharith F. M. Aljuwaie R. A. et al. (2025). Efficacy and safety of vancomycin, linezolid, and ceftaroline in the treatment of methicillin-resistant *Staphylococcus aureus* (MRSA): A systematic review and meta-analysis. *Cureus* 17:e77949. 10.7759/cureus.77949 39996230 PMC11848488

[B14] Ebrahimi S. Bölgen U. M. G. Kayıran S. D. Çevikelli T. Boğa M. Aksu F. (2026). In vitro and in vivo evaluation of an oral microemulsion formulation of Centaurea lycopifolia Boiss. Et Kotschy extract for analgesic and anti-inflammatory effects in a carrageenan-induced model. *Inflammopharmacology* 34, 1587–1602. 10.1007/s10787-026-02124-6 41656469 PMC12996417

[B15] El-Demerdash A. S. Orady R. M. Matter A. A. Ebrahem A. F. (2023). An Alternative approach using nano-garlic emulsion and its synergy with antibiotics for controlling biofilm-producing multidrug-resistant *Salmonella* in chicken. *Indian J. Microbiol.* 63, 632–644. 10.1007/s12088-023-01124-2 38034905 PMC10682320

[B16] El-Sherbiny G. M. Kalaba M. H. Foda A. M. Youssef A. S. E.-D. Elsehemy I. et al. (2024). Nanoemulsion of cinnamon oil to combat colistin-resistant *Klebsiella pneumoniae* and cancer cells. *Microb. Pathog.* 192:106705. 10.1016/j.micpath.2024.106705 38761892

[B17] Elwakil B. H. Shaaban M. M. Bakr B. A. Hamed M. T. Raj Paudel K. Atia A. M. et al. (2026). Novel eugenol/limonene nanoplatform as a new remedy against bacterial lung infections. *Sci. Rep.* 16:7728. 10.1038/s41598-026-38114-5 41741515 PMC12949021

[B18] Esen Yigit Ö Lamprecht A. (2026). Impact of drug hydrophilicity on transdermal delivery by nanoemulsions. *Pharmaceutics* 18:220. 10.3390/pharmaceutics18020220 41754962 PMC12944353

[B19] Franklyne J. S. Andrew Ebenazer L. Mukherjee A. Natarajan C. (2019). Cinnamon and clove oil nanoemulsions: Novel therapeutic options against vancomycin intermediate susceptible *Staphylococcus aureus*. *Appl. Nanosci.* 9, 1405–1415. 10.1007/s13204-019-01111-4

[B20] Gholizadeh P. Rahimzadeh R. Mohammadi-Ghalehbin B. Arzanlou M. Soozangar N. Feizi H. (2026). Enhanced antibiofilm and gene-suppressive effects of cinnamon nanoemulsion against multidrug-resistant *Enterococcus faecalis*. *Mol. Biol. Rep.* 53:419. 10.1007/s11033-026-11590-8 41733704

[B21] Ghosh V. Raza M. A. Ajazuddin. (2026). Optimising process parameters for development of basil (*Ocimum basilicum*) oil-based food-grade antibacterial oil-in-water microemulsion. *Flavour Fragr. J.* 41, 324–332. 10.1002/ffj.70043

[B22] Gopikrishnan M. Pramanick D. Poornima K. B. S. Gutam N. Doss C. Chandrasekaran N. (2026). Eugenol microemulsions exhibit potent activity against drug-resistant diabetic foot ulcer pathogens: Toward nanocomposite-based wound healing applications. *Microb. Pathog.* 218:108674. 10.1016/j.micpath.2026.108674 42385850

[B23] Haq N. Shahid M. Alaofi A. L. Ahmad Z. H. Alrayyes Y. F. Alsarra I. A. et al. (2023). Evaluation of the physicochemical and antimicrobial properties of nanoemulsion-based polyherbal mouthwash. *ACS Omega* 8, 41755–41764. 10.1021/acsomega.3c06176 37970055 PMC10634260

[B24] Hashem A. H. Doghish A. S. Ismail A. Hassanin M. M. H. Okla M. K. Saleh I. A. et al. (2024). A novel nanoemulsion based on clove and thyme essential oils: Characterization, antibacterial, antibiofilm and anticancer activities. *Electron. J. Biotechnol.* 68, 20–30. 10.1016/j.ejbt.2023.12.001

[B25] Hassan M. A. Noor S. Park J. Nabawy A. Dedhiya M. Patel R. et al. (2025). Gelatin nanoemulsion-based co-delivery of terbinafine and essential oils for treatment of *Candida albicans* biofilms. *Microorganisms* 13:127. 10.3390/microorganisms13010127 39858895 PMC11767362

[B26] Hayat M. Nawaz A. Ullah M. Latif M. S. Almuhayawi M. S. Zengin G. et al. (2025). Synthesis and formulation design of Sideroxylon mascatense branches extract mediated nano-emulsion cream: Characterization, antimicrobial, wound healing and antidiabetic activities assessment. *Waste Biomass Valorization* 16, 6529–6545. 10.1007/s12649-024-02779-8

[B27] Hu Q. Peng H. Rao X. (2016). Molecular events for promotion of vancomycin resistance in vancomycin intermediate *Staphylococcus aureus*. *Front. Microbiol.* 7:1601. 10.3389/fmicb.2016.01601 27790199 PMC5062060

[B28] Hughes C. S. Longo E. Phillips-Jones M. K. Hussain R. (2017). Characterisation of the selective binding of antibiotics vancomycin and teicoplanin by the VanS receptor regulating type A vancomycin resistance in the enterococci. *Biochim. Biophys. Acta BBA - Gen. Subj.* 1861, 1951–1959. 10.1016/j.bbagen.2017.05.011 28511809 PMC5482315

[B29] Izadiyan Z. Webster T. J. Kia P. Kalantari K. Misran M. Rasouli E. et al. (2025). Nanoemulsions based therapeutic strategies: Enhancing targeted drug delivery against breast cancer cells. *Int. J. Nanomedicine* 20, 6133–6162. 10.2147/IJN.S488545 40385492 PMC12085896

[B30] Jacob S. Kather F. S. Boddu S. H. S. Shah J. Nair A. B. (2024). Innovations in nanoemulsion technology: Enhancing drug delivery for oral, parenteral, and ophthalmic applications. *Pharmaceutics* 16:1333. 10.3390/pharmaceutics16101333 39458662 PMC11510719

[B31] Jadhav S. T. Salunkhe V. R. Bhinge S. D. (2023). Nanoemulsion drug delivery system loaded with imiquimod: A QbD-based strategy for augmenting anti-cancer effects. *Future J. Pharm. Sci.* 9:120. 10.1186/s43094-023-00568-z

[B32] Jahromy M. H. Qomi M. Fazelipour S. Sami N. Faali F. Karimi M. et al. (2024). Evaluation of curcumin-based ophthalmic nano-emulsion on atropine-induced dry eye in mice. *Heliyon* 10:e29009. 10.1016/j.heliyon.2024.e29009 38601632 PMC11004198

[B33] Jose A. Anitha Sasidharan S. Chacko C. Mukkumkal Jacob D. Edayileveettil Krishnankutty R. (2022). Activity of clove oil and chitosan nanoparticles incorporated PVA nanocomposite against *Pythium aphanidermatum*. *Appl. Biochem. Biotechnol.* 194, 1442–1457. 10.1007/s12010-021-03709-3 34739704

[B34] Kefayat A. Hamidi Farahani R. Rafienia M. Hazrati E. Hosseini Yekta N. (2022). Synthesis and characterization of cellulose nanofibers/chitosan/cinnamon extract wound dressing with significant antibacterial and wound healing properties. *J. Iran. Chem. Soc.* 19, 1191–1202. 10.1007/s13738-021-02374-x

[B35] Kim R. J. Park H. B. (2026). Cytoprotective effects of lipid emulsion against bupivacaine-induced cytotoxicity in human rotator cuff fibroblasts. *Antioxidants* 15:447. 10.3390/antiox15040447 42072089 PMC13113884

[B36] Kolluru G. K. Bir S. C. Kevil C. G. (2012). Endothelial dysfunction and diabetes: Effects on angiogenesis, vascular remodeling, and wound healing. *Int. J. Vasc. Med.* 2012:918267. 10.1155/2012/918267 22611498 PMC3348526

[B37] Kot B. Sytykiewicz H. Sprawka I. Witeska M. (2020). Effect of manuka honey on biofilm-associated genes expression during methicillin-resistant *Staphylococcus aureus* biofilm formation. *Sci. Rep.* 10:13552. 10.1038/s41598-020-70666-y 32782291 PMC7419495

[B38] Llarrull L. I. Fisher J. F. Mobashery S. (2009). Molecular basis and phenotype of methicillin resistance in *Staphylococcus aureus* and insights into New β-lactams that meet the challenge. *Antimicrob. Agents Chemother.* 53, 4051–4063. 10.1128/aac.00084-09 19470504 PMC2764218

[B39] Long Y. Zhang M. Yang C. (2024). Effects of flaxseed gum/egg white protein-based edible curcumin nanoemulsion coatings on starch retrogradation and quality of fried rice in freeze-thaw treatment. *Food Biosci.* 59:103977. 10.1016/j.fbio.2024.103977

[B40] Lorian V. (2005). *Antibiotics in Laboratory Medicine.* Philadelphia: Lippincott Williams & Wilkins.

[B41] Luo Q. Rong X. Xiao Z. Duan X. Zhou Y. Zhang J. et al. (2025). Effect of chitosan films containing clove essential oil-loaded microemulsions combined with deep learning on pork preservation and freshness monitoring. *Food Control* 168:110914. 10.1016/j.foodcont.2024.110914

[B42] Lwin N. T. Salahong T. Phongsopitanun W. Sripreechasak P. Chaotham C. Lertnitikul N. et al. (2026). Preparation, characterization and evaluation of microemulsions containing *Clausena excavata* Burm. f. leaf essential oil. *BMC Complement. Med. Ther.* 26:110. 10.1186/s12906-026-05300-x 41699583 PMC13015138

[B43] Mahor A. K. Singh P. P. Gupta R. Bhardwaj P. Rathore P. Kishore A. et al. (2023). Nanostructured lipid carriers for improved delivery of therapeutics via the oral route. *J. Nanotechnol.* 2023, 1–35. 10.1155/2023/4687959

[B44] Manzoor A. Asif M. Khalid S. H. Ullah Khan I. Asghar S. (2023). Nanosizing of lavender, basil, and clove essential oils into microemulsions for enhanced antioxidant potential and antibacterial and antibiofilm activities. *ACS Omega* 8, 40600–40612. 10.1021/acsomega.3c05394 37929152 PMC10621020

[B45] Martín S. A. Pérez I. Rivera A. (2021). Hosting of the antibiotic Vancomycin by bentonite: Characterization and slow release study. *Appl. Clay Sci.* 202:105965. 10.1016/j.clay.2020.105965

[B46] Meades G. Henken R. Waldrop G. Rahman M. D. Gilman S. Kamatou G. et al. (2010). Constituents of cinnamon inhibit bacterial acetyl CoA carboxylase. *Planta Med.* 76, 1570–1575. 10.1055/s-0030-1249778 20379951

[B47] Miranda-Calderon L. Yus C. Landa G. Mendoza G. Arruebo M. Irusta S. (2022). Pharmacokinetic control on the release of antimicrobial drugs from pH-responsive electrospun wound dressings. *Int. J. Pharm.* 624:122003. 10.1016/j.ijpharm.2022.122003 35811042

[B48] Nagaiah H. P. Samsudeen M. B. Augustus A. R. Shunmugiah K. P. (2025). In vitro evaluation of silver-zinc oxide-eugenol nanocomposite for enhanced antimicrobial and wound healing applications in diabetic conditions. *Discov. Nano* 20:14. 10.1186/s11671-025-04183-0 39847138 PMC11757845

[B49] Osanloo M. Noori F. Tavassoli A. Ataollahi M. R. Davoodi A. Seifalah-Zade M. et al. (2023). Effect of PCL nanofiber mats coated with chitosan microcapsules containing cinnamon essential oil for wound healing. *BMC Complement. Med. Ther.* 23:84. 10.1186/s12906-023-03905-0 36934283 PMC10024394

[B50] Oun A. A. Shin G. H. Kim J. T. (2022). Multifunctional poly(vinyl alcohol) films using cellulose nanocrystals/oregano and cellulose nanocrystals/cinnamon Pickering emulsions: Effect of oil type and concentration. *Int. J. Biol. Macromol.* 194, 736–745. 10.1016/j.ijbiomac.2021.11.119 34838863

[B51] Parın F. N. El-Ghazali S. Yeşilyurt A. Parın U. Ullah A. Khatri M. et al. (2023). PVA/inulin-based sustainable films reinforced with pickering emulsion of niaouli essential oil for potential wound healing applications. *Polymers* 15:1002. 10.3390/polym15041002 36850285 PMC9966936

[B52] Raeisi M. Tajik H. Yarahmadi A. Sanginabadi S. (2015). Antimicrobial effect of cinnamon essential oil against *Escherichia Coli* and *Staphylococcus aureus*. *Health Scope* 4:e21808. 10.17795/jhealthscope-21808

[B53] Ragupathi N. K. D. Muthuirulandi Sethuvel D. P. Gopikrishnan M. Dwarakanathan H. T. Murugan D. Biswas I. et al. (2023). Phage-based therapy against biofilm producers in gram-negative ESKAPE pathogens. *Microb. Pathog.* 178:106064. 10.1016/j.micpath.2023.106064 36898591

[B54] Ribeiro R. Rentes B. R. Honorato L. A. Kuhnen S. (2024). Effect of nanoemulsion loaded with macela (*Achyrocline satureioides*) on the ultrastructure of *Staphylococcus aureus* and the modulating activity of antibiotics. *Front. Nanotechnol.* 6:1466988. 10.3389/fnano.2024.1466988

[B55] Salama A. Qushawy M. Elsherbiny N. Hetta H. F. Alqifari S. F. Safwat M. A. et al. (2026). Myrrh oil-based nanoemulsion loaded with curcumin and insulin: Development, characterization, and evaluation of enhanced antibacterial and diabetic wound-healing activity. *Pharmaceutics* 18:369. 10.3390/pharmaceutics18030369 41900855 PMC13030445

[B56] Sarhan O. M. Abdelqader S. O. Mostafa M. S. A. Badawi M. A. E. Ahmed M. A. M. El Sayed W. G. A. et al. (2025). Exploring the antidiabetic effect of various therapeutic essential oils synergistically with repaglinide. *J. Pharm. Sci.* 114:103721. 10.1016/j.xphs.2025.103721 40058589

[B57] Scalia A. C. Talamo Ruiz J. A. Dalle Vacche S. Bongiovanni R. Lacroix-Desmazes P. Cochis A. et al. (2026). Multifunctional eugenol-enriched PEO-based electrospun and photo-crosslinked scaffolds for wound healing applications. *Appl. Mater. Today* 49:103136. 10.1016/j.apmt.2026.103136

[B58] Seifi M. Esfandiary M. A. Hemmati N. Malakootikhah J. Nikaein D. Khosravi A. R. (2026). Nano-encapsulated *Cinnamomum cassia* essential oil in combination with fluconazole exhibits synergistic activity against fluconazole-resistant *Nakaseomyces glabratus* and *Pichia kudriavzevii*. *Vet. Med. Sci.* 12:e71001. 10.1002/vms3.71001 42157708 PMC13239926

[B59] Selim S. (2022). Mechanisms of gram-positive vancomycin resistance (Review). *Biomed. Rep.* 16, 1–6. 10.3892/br.2021.1490 34938536 PMC8686198

[B60] Shaghayegh G. Cooksley C. Cheney C. Rad S. K. Panchatcharam B. S. Barry E. F. et al. (2026). A dual setback: *Staphylococcus aureus* biofilms from severe chronic rhinosinusitis show enhanced virulence capacity and tolerance to antibiotics. *Int. Forum Allergy Rhinol.* 16, 541–552. 10.1002/alr.70092 41493114

[B61] Sharma H. K. Singh I. Karna A. Gupta P. Singh T. Kumar A. et al. (2025). Counteracting methicillin resistant *Staphylococcus aureus* through novel Citral-Azithromycin combination. *Sci. Rep.* 15:25548. 10.1038/s41598-025-11721-4 40664725 PMC12263860

[B62] Shi Q. Zhao R. Wang J. Xiong Z. Deng X. Zheng C. et al. (2026). Photothermal–immunomodulatory hydrogel reinforced by Ti3C2/ZnAl-LDH nanoplatform for eradicating MRSA and promoting diabetic wound healing. *Adv. Sci.* 13:e17577. 10.1002/advs.202517577 41556379 PMC13042765

[B63] Singh S. Datta S. Narayanan K. B. Rajnish K. N. (2021). Bacterial exo-polysaccharides in biofilms: Role in antimicrobial resistance and treatments. *J. Genet. Eng. Biotechnol.* 19:140. 10.1186/s43141-021-00242-y 34557983 PMC8460681

[B64] Sorenson T. R. Zack K. M. Joshi S. G. (2025). Biofilm formation and the role of efflux pumps in ESKAPE pathogens. *Microorganisms* 13:1816. 10.3390/microorganisms13081816 40871320 PMC12388334

[B65] Sosa A. M. Cottet C. Berin B. E. Martínez L. M. Peltzer M. A. Prieto M. J. et al. (2025). Development of films for wound healing based on gelatin and oil/water emulsions as carriers of bioactive compounds. *Pharmaceutics* 17:357. 10.3390/pharmaceutics17030357 40143021 PMC11944759

[B66] Sultana R. Ahmed I. Saima S. Salam M. T. Sultana S. (2023). Diabetic foot ulcer-a systematic review on relevant microbial etiology and antibiotic resistance in Asian countries. *Diabetes Metab. Syndr. Clin. Res. Rev.* 17:102783. 10.1016/j.dsx.2023.102783 37257221

[B67] Tabaniag J. S. U. Abad M. Q. D. Morcelos C. J. R. Geraldino G. V. B. Alvarado J. L. M. Lopez E. C. R. (2023). Stabilization of oil/water emulsions using soybean lecithin as a biobased surfactant for enhanced oil recovery. *J. Eng. Appl. Sci.* 70:154. 10.1186/s44147-023-00322-5

[B68] Tambe E. Gotmare S. (2022). Ftir analysis and interpretation of ir spectra of four spice oils extracted by hydrodistillation. *World J. Pharm. Pharm. Sci.* 11, 1346–1363.

[B69] Thanakarn W. Torres-Acosta M. A. Dikicioglu D. (2026). Reducing the sensitivity of Halomonas sp. to oxygen availability through adaptive laboratory evolution. *Microb. Cell Factories* 25:74. 10.1186/s12934-026-02951-w 41680775 PMC12997722

[B70] Trindade G. A. de M. Alves L. A. Lazo R. E. L. Dallabrida K. G. Reolon J. B. et al. (2024). Polysaccharide-stabilized semisolid emulsion with vegetable oils for skin wound healing: Impact of composition on physicochemical and biological properties. *Pharmaceutics* 16:1426. 10.3390/pharmaceutics16111426 39598549 PMC11597777

[B71] Tungadi R. (2024). The effect of ultrasonication time on particle size, polydispersity index and stability evaluation of anthocyanin liposomes. *Univers. J. Pharm. Res* 9, 8–13. 10.22270/ujpr.v9i1.1056

[B72] Wilkinson A.-S. Ward S. Kania M. Page M. G. P. Wharton C. W. (1999). Multiple conformations of the acylenzyme formed in the hydrolysis of methicillin by *Citrobacter freundii* β-lactamase:? A time-resolved FTIR spectroscopic study. *Biochemistry* 38, 3851–3856. 10.1021/bi990030i 10194295

[B73] Yang Z. Chai Y. Zhou D. Yao X. Ji H. (2019). Mechanism for efficient separation of eugenol and eugenol acetate with β -cyclodextrin as a selective solvent. *Supramol. Chem.* 31, 1–9. 10.1080/10610278.2019.1702663

[B74] Yazgan H. (2022). Antimicrobial activities of emulsion-based edible solutions incorporating lemon essential oil and sodium caseinate against some food-borne bacteria. *J. Food Sci. Technol.* 59, 4695–4705. 10.1007/s13197-022-05551-9 36276524 PMC9579266

[B75] Zafar S. Sattari-Maraji A. Moghadam S. O. Nejati A. Kharrazi S. Ahadi E. M. et al. (2026). Potential of Jelleine-I peptide on down-regulation of biofilm-associated genes and the biofilm formation of methicillin-resistant *Staphylococcus aureus*. *Mol. Biol. Rep.* 53:506. 10.1007/s11033-026-11640-1 41848960

[B76] Zhao X. Zhang Y. Huang Z. Wu X. Lin J. (2025). Innovative therapies for diabetic foot ulcers: Application and prospects of smart dressings. *Biomed. Pharmacother.* 191:118498. 10.1016/j.biopha.2025.118498 40882525

